# Age-time-specific transmission of hand-foot-and-mouth disease enterovirus serotypes in Vietnam: A catalytic model with maternal immunity

**DOI:** 10.1016/j.epidem.2024.100754

**Published:** 2024-03

**Authors:** Yining Chen, Lam Anh Nguyet, Le Nguyen Thanh Nhan, Phan Tu Qui, Le Nguyen Truc Nhu, Nguyen Thi Thu Hong, Nguyen Thi Han Ny, Nguyen To Anh, Le Kim Thanh, Huynh Thi Phuong, Nguyen Ha Thao Vy, Nguyen Thi Le Thanh, Truong Huu Khanh, Nguyen Thanh Hung, Do Chau Viet, Nguyen Tran Nam, Nguyen Van Vinh Chau, H. Rogier van Doorn, Le Van Tan, Hannah Clapham

**Affiliations:** a Saw Swee Hock School of Public Health, National University of Singapore and National University Health System, Singapore; b Oxford University Clinical Research Unit, Ho Chi Minh City, Viet Nam; c Children’s Hospital 1, Ho Chi Minh City, Viet Nam; d Hospital for Tropical Diseases, Ho Chi Minh City, Viet Nam; e Children’s Hospital 2, Ho Chi Minh City, Viet Nam; f City Children’s Hospital, Ho Chi Minh City, Viet Nam; g Oxford University Clinical Research Unit, Hanoi, Viet Nam; h Centre for Tropical Medicine and Global Health, Nuffield Department of Clinical Medicine, University of Oxford, Oxford, United Kingdom

**Keywords:** Hand, foot and mouth disease, Serological data, Catalytic model, Force of infection

## Abstract

Hand, foot and mouth disease (HFMD) is highly prevalent in the Asia Pacific region, particularly in Vietnam. To develop effective interventions and efficient vaccination programs, we inferred the age-time-specific transmission patterns of HFMD serotypes enterovirus A71 (EV-A71), coxsackievirus A6 (CV-A6), coxsackievirus A10 (CV-A10), coxsackievirus A16 (CV-A16) in Ho Chi Minh City, Vietnam from a case data collected during 2013–2018 and a serological survey data collected in 2015 and 2017. We proposed a catalytic model framework with good adaptability to incorporate maternal immunity using various mathematical functions. Our results indicate the high-level transmission of CV-A6 and CV-A10 which is not obvious in the case data, due to the variation of disease severity across serotypes. Our results provide statistical evidence supporting the strong association between severe illness and CV-A6 and EV-A71 infections. The HFMD dynamic pattern presents a cyclical pattern with large outbreaks followed by a decline in subsequent years. Additionally, we identify the age group with highest risk of infection as 1-2 years and emphasise the risk of future outbreaks as over 50% of children aged 6-7 years were estimated to be susceptible to CV-A16 and EV-A71. Our study highlights the importance of multivalent vaccines and active surveillance for different serotypes, supports early vaccination prior to 1 year old, and points out the potential utility for vaccinating children older than 5 years old in Vietnam.

## Highlights

• We proposed a modified catalytic model framework with maternal immunity.
• We made use jointly of case and serology data to estimate transmission intensity.
• The results reveal the high-level transmission of coxsackievirus A6, A10 in Vietnam.
• The results identify the age with highest infection risk as 1-2 years old.
• This study emphasises the importance of multivalent vaccines and active surveillance.

## 1 Introduction

Hand, foot and mouth disease (HFMD), a common infectious disease mainly appearing among children under 5 years old, is a disease with high burden in Asia-Pacific area (Puenpa et al., 2019, Gonzalez et al., 2019). Vietnam is a Southeast Asian country that has experienced large HFMD outbreaks in recent years since 2005, with enterovirus A71 (EV-A71), coxsackievirus A6 (CV-A6), coxsackievirus A10 (CV-A10), coxsackievirus A16 (CV-A16) as the virus serotypes causing majority of the reported cases (Van Hoang et al., 2019, Thanh Nhan et al., 2020, Tu et al., 2007). HFMD spreads in Vietnam with a seasonal pattern where climate factors such as temperature and humidity have shown substantial association with the dynamic pattern of HFMD (Nguyen et al., 2020). The symptoms of HFMD infections vary among different infected virus serotypes with individual level variation (Van Hoang et al., 2019). Children infected by HFMD viruses may experience asymptomatic infection, mild symptoms or suffer from severe illness requiring hospitalisation or even causing death (Mirand et al., 2012, Zhao et al., 2020, Esposito and Principi, 2018). In particular, analysis about clinical presentation among hospitalised infected children found strong association between EV-A71 and severe symptoms (Van Hoang et al., 2019, Thanh Nhan et al., 2020). These understandings of the disease are useful in guiding interventions and policies controlling the disease and improving public health under the burden of HFMD.

For effective control the spread of HFMD, vaccine development is the crucial strategy. There are three approved and marketed inactivated monovalent vaccines targeting EV-A71 available in mainland China, and there is an inactivated EV-A71 vaccine developed by a Taiwanese company currently completing phase 3 clinical trials (Zhu et al., 2013, Zhu et al., 2014, Li et al., 2014, Nguyen et al., 2022). The vaccination program in mainland China targets children aged 6 months to 5 years old and recommends getting vaccination before 12 months old (Chinese Center for Disease Contro and Prevention, 2016) while the Taiwanese vaccine covers children aged <6 months. Currently, the state-of-art direction of HFMD vaccine development is multivalent vaccines, most of which are in animal testing research phase (Zhang et al., 2023). Understanding the transmission patterns of HFMD serotypes can yield valuable insights for determining the serotypes that the vaccine should prioritise targeting. The age-structure spread pattern of HFMD is also important in guiding the implementation of an effective vaccine program for the region. Specifically, knowledge about disease dynamic patterns of different age groups not only works as a useful guideline for vaccination target population and efficient vaccination age, but also illustrates the disease dynamic pattern throughout the lifespan at population level, provides insights for controlling and preventing HFMD outbreaks. However, there is limited research about the age-varying transmission patterns of HFMD viruses in South East Asia.

Two widely used data sources including information about disease spread patterns are serological survey data and case data from clinics, hospital wards or surveillance systems. Case data sets are easier to obtain, more convenient to interpret and directly revealing the dynamic pattern of the disease. However, the actual dynamics of the disease may be masked by the variation in disease severity of cases infected by different virus serotypes. The susceptible proportion of the population, which is a useful measurement warning potential outbreaks and identifying vaccine target population, is indirectly presented in case data sets. In contrast, though lacking direct reflection of case patterns, serological survey data sets provide straightforward information about seroprevalence, susceptible proportion of the population and infection history including asymptomatic infections. In recent years, HFMD case data in Vietnam has been used to analyse HFMD symptom variation across different infected serotypes, the phylogenic patterns of emerging cases, frequency of cases infected by different virus serotypes, and the association between disease outbreaks and climate factors (Van Hoang et al., 2019, Thanh Nhan et al., 2020, Hoa-Tran et al., 2020, Nguyen et al., 2020). Serological survey data of HFMD in Vietnam has been used to analyse seroprevalence of EV-A71 and maternal immunity (Nguyen Tran et al., 2011, Kuo et al., 2020). Despite the differences between case data and serological survey data, both can provide information about the transmission patterns of HFMD viruses. However, most studies made use of only one of these two types of data to extract different information. Mathematical model frameworks applicable for both case data and serological survey data are capable of gathering information from multiple data sources and improving the efficiency of data analysis.

As a fundamental model framework utilised to infer virus transmission patterns from case data and serological survey data, catalytic model has been widely applied to surveillance data recording age-stratified cases of infectious diseases such as HFMD, dengue, and Japanese encephalitis, demonstrating high efficacy (Zhao et al., 2016, Le et al., 2022, Rodriguez-Barraquer et al., 2019, Quan et al., 2020). Meanwhile, the catalytic model is popular in estimating time-varying or age-varying infection risk from serological survey data for example for dengue and chikungunya (Nealon et al., 2022, Lam et al., 2019, Kumar et al., 2021). However, the general catalytic model lacks flexible structure of incorporating maternal immunity. Previous studies included maternal immunity under the catalytic model framework by using the analytic function of Susceptible-Infectious-Recovered (SIR) model with maternal immunity or assuming a delay of infection with pre-fixed maternal immunity waning length (Pons-Salort et al., 2023, Zhao et al., 2016). For HFMD, there is little research applying the models assuming the existence of maternal immunity while maternal immunity of HFMD is supported by various serological evidence from previous studies (Nguyen Tran et al., 2011, Zhou et al., 2022, Fu et al., 2016, Yang et al., 2022). Given that HFMD is a disease primarily infecting children, neglecting maternal immunity during modelling may introduce bias to the estimations. Besides, in the research area of modelling for different infectious diseases including HFMD, majority of studies inferred either time-varying infection risk or age-varying infection risk. There is limited research making age-time-specific transmission pattern inference, which can provide more comprehensive information about the disease spread pattern.

In this study, we made use of the case data collected from 2013 to 2018 with age information and the serological survey data collected in 2015 and 2017 for children with different ages. We aimed to infer age-time-specific transmission patterns of 4 HFMD virus serotypes: CV-A6, CV-A10, CV-A16, EV-A71 in Ho Chi Minh City, Vietnam with consideration of maternal immunity. Our study can make efficient use of the data sets, draw a thorough picture of the age-time-varying disease dynamic pattern, and provide helpful guidance for developing effective interventions and vaccine strategies to control and prevent HFMD outbreaks. The mathematical models are also flexible with different assumptions of maternal immunity and useful for other infectious diseases.

## 2 Results

### 2.1 Model fitting evaluation

To infer comprehensive transmission patterns of HFMD in Ho Chi Minh City, Vietnam, we modified the catalytic model framework to incorporate maternal immunity, applied serotype-varying scaling factors to account for the difference between actual infections and hospitalised cases, and made joint use of the case data and serological survey data. The case data includes 1,772 HFMD patients aged <12 years old infected by CV-A6, CV-A10, CV-A16, EV-A71. The patients were recruited from Children’s Hospital 1, Children’s Hospital 2, and Hospital for Tropical Diseases in Ho Chi Minh City, Vietnam from 2013 to 2018. The serological survey data includes 48 residual serum samples collected from subjects at the Hospital for Tropical Diseases in 2015 and 52 residual serum samples collected in 2017. The serological data includes 2 individuals aged less than 1 year old, with ages as 0.77 years and 0.80 years respectively (see Supplementary Table 1 for sample size by year and age in the serological survey data). We applied 4 catalytic models assuming maternal immunity with different model settings where age-time-specific Force of Infection (FOI) was estimated under Bayesian model framework. The 4 models are named as ExpPw, ExpFt, LinPw, LinFt where Exp refers to models with maternal immunity described by exponential functions, Lin refers to models with maternal immunity described by linear functions, Pw refers to models with FOI constructed by piecewise function, Ft refers to models with FOI constructed by Farrington’s function. (Details in Materials and methods and Supplementary Methods).

The posterior distributions of parameters in Bayesian models were estimated by generating Hamiltonian Monte Carlo (HMC) samples. Our 4 models converged well with the minimum effective sample size larger than 3,000 and the maximum R hat value smaller than 1.0009 (see Supplementary Table 2). The HMC chains also mixed well as shown in the trace plots (Supplementary Figure 1–4). The models ranked from smallest Deviance Information Criterion (DIC) to largest DIC are LinFt (DIC: 1,715), LinPw (DIC: 1,717), ExpFt (DIC: 1,782), ExpPw (DIC: 1,785) where smaller DIC indicates that the model fits the data better (Spiegelhalter et al., 2002). The validity of DIC was substantiated by the number of effective parameters (see Supplementary Results). This implies that the models with maternal immunity described by linear functions (Model LinFt, LinPw) perform better with considerably smaller DIC than that with exponential functions (Model ExpFt, ExpPw) while models with FOI constructed by Farrington’s function and piecewise function have similar model fitting effect. The model fitting effect was visualised by comparing the observed number of cases (Supplementary Fig. 5) and seroprevalence (Supplementary Fig. 6) to the ones predicted by the 4 models. 4 models make similar estimations of the number of cases, all of which match the case data well. In contrast, though exhibiting similar age-time-varying dynamic patterns, the four models show a greater variation in estimations for seroprevalence compared to case numbers, especially for serotypes CV-A16 and EV-A71.

**Fig. 1:**
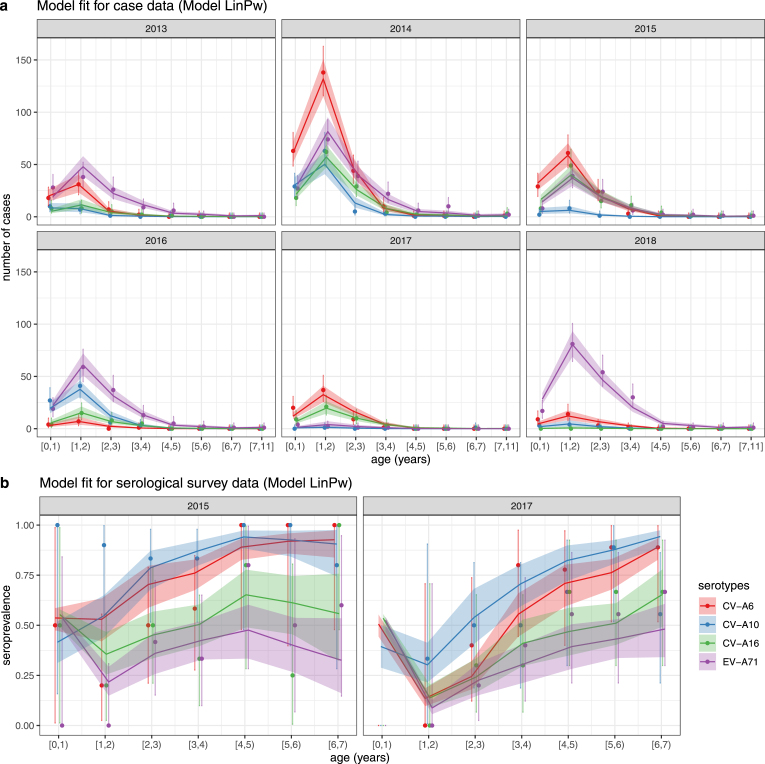
The reported number of cases by age and calendar year in the case data and from the model estimation (Model LinPw) and the seroprevalence (seropositive threshold titre of 1:8) by age and calendar year in the serological survey data and from the model estimation (Model LinPw). a. The age-time-specific reported number of cases in case data and the corresponding estimation. b. The age-time-specific seroprevalence in serological survey data and the corresponding estimation. The solid points and the error bars are the data points and the 95% confidence intervals calculated by exact method (Liddell, 1984, Hirji, 2005) (no solid points for age group [0,1) in b. for 2017 panel because the serological survey data does not include subjects with age <1 year old collected in 2017). The lines and the coloured areas are the estimated values and the 95% confidence intervals inferred from posterior distributions of the parameters. The colours correspond to 4 serotypes. (For interpretation of the references to colour in this figure legend, the reader is referred to the web version of this article.)

As piecewise function relies on simpler assumption of FOI than Farrington’s function, and the models describing maternal immunity with linear functions perform better than that with exponential functions in terms of model fitting effect, the results presented in the remainder of this section are from the catalytic model with linear functions for maternal immunity and piecewise function for FOI (Model LinPw) unless stated otherwise. As shown in Fig. 1 comparing the data observation and model predictions, the model exhibits good fit on case data as the estimated distribution of the reported number of cases closely matches that of the data. While there is a slight mismatch between the estimated seroprevalence and the serological data for the young age groups 0–1 and 1–2 years, this difference could potentially be attributed to the wide confidence intervals of the data points, i.e., the small sample size. Despite the minor discrepancy, the estimation of seroprevalence effectively captures the underlying pattern in the serological survey data.

### 2.2 Force of Infection and HFMD disease severity estimates

**Fig. 2:**
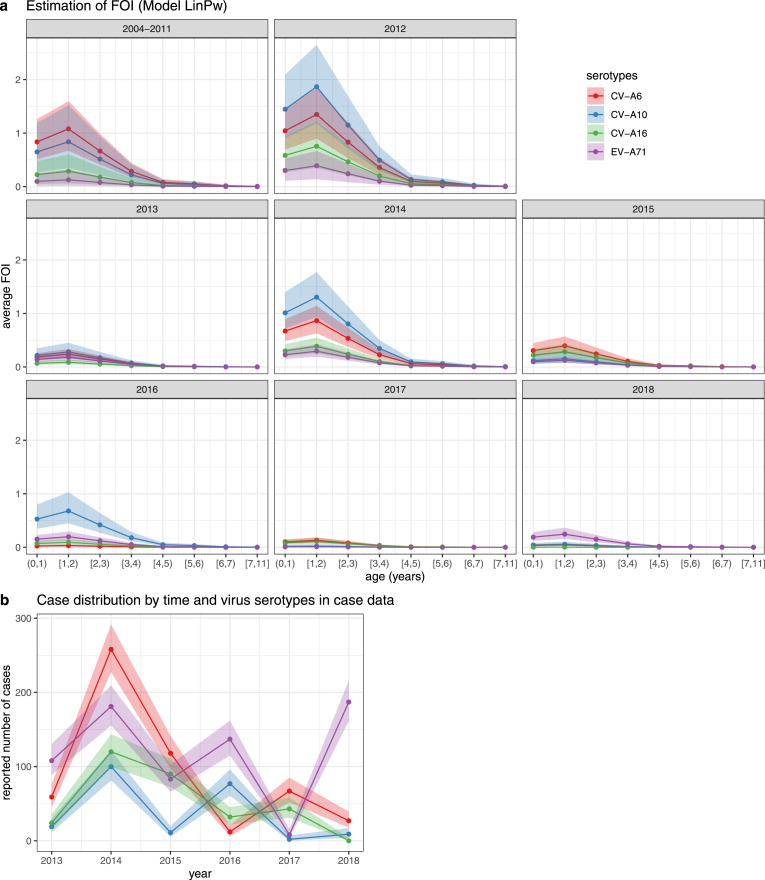
The estimation of age-time-specific FOI from Model LinPw and the distribution of reported cases in case data by time. a. The estimation of FOI. The solid points are the estimates. The coloured areas are the 95% confidence intervals inferred from posterior distributions of the parameters. The colours correspond to 4 serotypes. b. The reported number of cases by time and virus serotypes in case data. The solid points are the data points. The coloured areas are the 95% confidence intervals calculated by exact method (Liddell, 1984). The colours correspond to 4 serotypes. (For interpretation of the references to colour in this figure legend, the reader is referred to the web version of this article.)

As shown in Fig. 2, children of age 1 to 2 years without immune protection were most likely to be infected because the estimated FOI peaked at age 1–2 years old. Additionally, Ho Chi Minh City experienced a high-level transmission of HFMD in 2004–2011, 2012 and 2014 with relatively high estimated FOI, which is always interpreted as risk of infection (Eq. (5)). In particular, the estimated FOI changed in a cyclical pattern by time where the overall transmission magnitude was relatively high in years 2004–2011 (highest risk of infection: 0.66, 95% confidence interval (CI): 0.50−0.80), 2012 (0.85, 95% CI: 0.69−0.93), 2014 (0.73, 95% CI: 0.61−0.83), 2016 (0.49, 95% CI: 0.36−0.64), 2018 (0.22, 95% CI: 0.14−0.31), followed by a decline in the subsequent year 2013 (0.25, 95% CI: 0.15−0.36), 2015 (0.33, 95% CI: 0.23−0.44), 2017 (0.12, 95% CI: 0.09−0.17). The FOI estimation is more sensitive to the functions describing maternal immunity than the functions constructing FOI, particularly for serotypes CV-A16 and EV-A71 with maximum fluctuation at around 0.1, i.e., fluctuation of infection risk at around 0.08 (Supplementary Fig. 7). Despite minor variation in the FOI estimations across four models, the pattern of relative changes in FOI across different age groups and over time remained similar. The hospitalised infections as shown in Fig. 2b also presented a cyclical pattern for both the overall HFMD spread magnitude and the dynamic patterns varied by serotypes. Particularly, CV-A6 and CV-A16 showed a 2-to-3-year cyclical pattern while CV-A10 and EV-A71 showed a 2-year cyclical pattern for the hospitalised cases.

The estimation of FOI also suggests the high-level transmission of serotypes CV-A6 and CV-A10, which were estimated to be the predominant virus serotypes during the study period except in 2018. More specifically, CV-A10 was the predominant virus serotype in 2012, 2013, 2014 and 2016; CV-A6 was the predominant virus serotype in 2004–2011, 2015 and 2017. In contrast, we estimated that CV-A16 and EV-A71 had a low-level transmission during the study period except in 2015, 2017 and 2018, when the overall spread magnitude of HFMD was also low. Specifically, CV-A16 was the predominant virus serotype in 2017 circulating with CV-A6; EV-A71 was the predominant virus serotype in 2018 while the other virus serotypes had risk of infection close to 0 in 2018.

The estimated low-level transmission of EV-A71 and high-level transmission of CV-A10 are not obviously revealed in the case data (Figs. 1a, 2b) where the difference was estimated by the serotype-varying scaling factors (Details in Materials and methods). Large HFMD outbreaks caused by EV-A71 have been observed in 2011–2012 and 2018 by the hospitalised cases in the case data and previous research (Thu Hong et al., 2018). However, large hospitalisations are not directly correlated with high-level transmission and vice versa, which can be attributed to the variation of disease severity related to different virus serotypes (Chen et al., 2012, Gaunt et al., 2015). We estimated that CV-A10 had the smallest scaling factor (0.0007, 95% CI: 0.0006−0.0009), which indicates the lowest probability of developing severe symptoms. By taking the scaling factor of CV-A10 as the reference, we calculated the relative scaling factor for CV-A6, CV-A16 and EV-A71. The relative scaling factor varied by serotypes ranked from highest (4.31) to lowest (2.32) is EV-A71 (4.31, 95% CI: 2.97−6.60), CV-A6 (3.17, 95% CI: 2.35−4.31), CV-A16 (2.32, 95% CI: 1.60−3.46) (see Supplementary Table 3 for estimations from other models). The highest scaling factor of EV-A71 indicates that the EV-A71-related infections have the largest probability of developing severe symptoms where the estimated scaling factor of EV-A71 is about 4.31 times that of CV-A10. As the serotypes with relatively stronger association with severe infections, EV-A71 and CV-A6 were responsible for majority of hospitalised cases during the study period though EV-A71 presented a low-level transmission pattern during 2004 to 2017 in the estimation of FOI. In contrast, CV-A10 and CV-A16, weakly associated with severe symptoms, caused a small amount of hospitalised cases despite the high-level transmission of CV-A10. Particularly, the relatively small number of CV-A16-related infections observed in the case data can be attributed to a combination effect of the low-level transmission pattern estimated by FOI and the weak association with severe symptoms. The estimation of FOI in our study can reveal the transmission patterns of HFMD viruses including silent transmission that may be obscured by the impact of serotypes on disease severity in case data.

### 2.3 Seroprevalence of HFMD

**Fig. 3:**
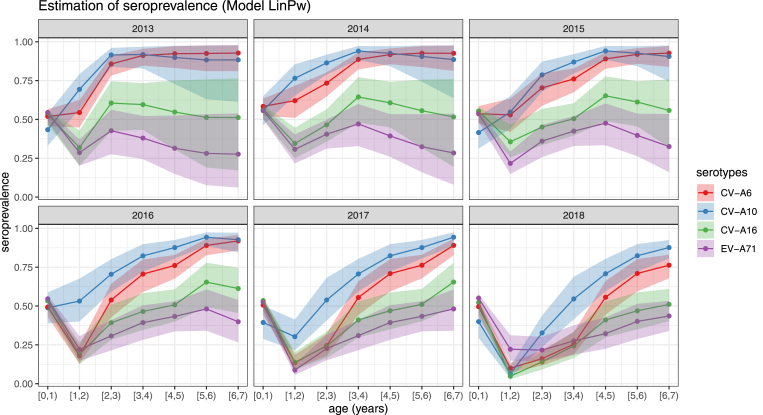
The estimation of seroprevalence by age, time and virus serotypes (Model LinPw). The solid points are the estimates. The coloured areas are the 95% confidence intervals inferred from posterior distributions of the parameters. The colours correspond to 4 serotypes. (For interpretation of the references to colour in this figure legend, the reader is referred to the web version of this article.)

Fig. 3 is the estimation of seroprevalence by age, time and virus serotypes (see Supplementary Fig. 6 for seroprevalence estimations from 4 models). The seroprevalence of CV-A6 and CV-A10 reached around 90% for children of age 6 to 7 years, indicating the high-level transmission of CV-A6 and CV-A10. In contrast, the seroprevalence of CV-A16 and EV-A71 was only around 50% for children aged 6–7 years. The seroprevalence of serotypes in different years presented similar age-varying change pattern. With the existence of maternal immunity, the seroprevalence was relatively high for infants aged 0 to 1 year and decreased by age as the maternal immunity disappeared. With cumulative immune protection acquired from natural infection, the seroprevalence gradually rose as age increased from 1–2 years old. The estimated large HFMD outbreak in 2011–12 as in Fig. 2a resulted in a high seroprevalence among the cohort affected by the outbreak especially for the children aged 1–2 years in 2012, whose seroprevalence of CV-A10 and CV-A6 reached a high level at around 94% and 89%. The slight decrease of seroprevalence by age after 1–2 years old can be attributed to this cohort, especially for serotypes CV-A16 and EV-A71. Furthermore, the estimated seroprevalence of EV-A71 was lowest among all analysed serotypes, highlighting population-level susceptibility where the low seroprevalence in 2017 might explain the EV-A71 outbreak in 2018. Specifically, the estimated EV-A71 seroprevalence for children aged 6 to 7 years remained at a low-level in 2013–2014 (28%, 95% CI: 8%−54%), 2015 (33%, 95% CI: 16%−54%), 2016 (40%, 95% CI: 27%−54%),2017–2018 (48%, 95% CI: 34%−61%). The observed pattern is evident in the seroprevalence estimations from 4 models, despite the minor difference.

### 2.4 Maternal immunity insights from modelling

We estimated that CV-A10 had larger decreasing rate of maternal immunity than other serotypes while the difference is not significant regarding the overlapping confidence intervals. The serotypes ranked from lowest to highest by the estimated age that all children lose maternal immunity ($1/\xi^{S}$ in Eq. (3)) were CV-A10 (0.69 years old, 95% CI: 0.48−0.91), CV-A6 (0.89 years old, 95% CI: 0.76−0.98), CV-A16 (0.95 years old, 95% CI: 0.83−0.99), EV-A71 (0.96 years old, 95% CI: 0.85−0.99). Despite the different mathematical descriptions of maternal immunity, our 4 models provide consistent statistical evidence about the order of maternal immunity decreasing rate across serotypes (Supplementary Table 4).

### 2.5 Simulation study and sensitivity test

We conducted a simulation study for Model LinPw to investigate the impact of sample size in serological survey data. The performance of the model at inferring FOI, the maternal immunity decline pattern and the serotype-specific scaling factor was evaluated using the 100 simulated case data alone as well as in combination with the 100 simulated serological data where there are 3 sets of simulated serological data having the he same sample size as the data used in this study or larger sample size (See Materials and methods for details). With case data alone, the model performs well at estimating FOI with average estimate close to the simulated value, estimate range covering the simulated value and coverage rate at around 80% to 100% (Supplementary Fig. 8,9). The incorporation of serological data obviously improves the model performance of inferring FOI with reduced uncertainty, as shown in the more accurate average estimate, narrower estimate range (Supplementary Fig. 8) and higher coverage rate at around 90% to 100% (Supplementary Fig. 9). Furthermore, the model has adequate ability to infer the maternal immunity decreasing pattern with average estimates closely matching the simulated value and coverage rate at around 95% to 100%, despite the relatively large uncertainty that the estimated range spans approximately 0.3 to 0.5 years (Supplementary Table 5). The model demonstrates relatively weaker performance in estimating the scaling factors with coverage rate at around 85% to 100% while the average estimate still matches the simulated value well (Supplementary Table 5). The inclusion of serological data improves the model performance at inferring scaling factor with lower uncertainty. Further increasing the sample size of the serological data does not significantly improve the model performance compared to that using jointly the case data and serological data with the same sample size as the actual data (Supplementary Fig. 8,9, Table 5).

There is limited research about the seroprevalence of antibodies against HFMD viruses among new-born infants or mothers in Ho Chi Minh City, Vietnam where there is only one paper reporting 55% seroprevalence against EV-A71 for infants (Nguyen Tran et al., 2011). In this study, we assumed that all infants born with maternal immunity and conducted a sensitivity test for 4 model structures LinFt, LinPw, ExpFt, ExpPw by assuming the proportion of children born with maternal immunity (denoted as q) as 50%,60%,…,90% and no maternal immunity. The model fitting effect was measured using DIC (see Supplementary Table 6 for the effective number of parameters) and by comparing estimations with data points for both case numbers and seroprevalence. The robustness of disease dynamics estimations was assessed by the fluctuation of estimated FOI.

Model LinPw is the model most robust to the proportion of children born with maternal immunity, as shown in the stable estimations of case number, seroprevalence (except the variation of seroprevalence for new-born infants due to the various assumption of q) and FOI (Supplementary Fig. 11–13). The proportion of children born with maternal immunity as 100% shows best model fitting effect with smallest DIC. The DIC becomes worse as the proportion decreases (Supplementary Fig. 10). In models LinFt, ExpFt, ExpPw, the estimation of case number for young age groups 0−1 year and 1−2 years is slightly sensitive to the choices of q; The FOI estimation is moderately sensitive to the selection of q for the dynamics in the first few years of life including age groups 0−1 year, 1−2 years and 2−3 years, with maximum fluctuation as around 0.3 (Supplementary Fig. 14–22). The estimations of seroprevalence are more sensitive to the proportion of children born with maternal immunity in models with FOI constructed by Farrington’s function than models with FOI constructed by piecewise function (Supplementary Fig. 12,15,18,21). Linear functions, estimating a faster decline in maternal immunity than exponential functions (Supplementary Fig. 23), aligns closer with the serological cohort study indicating complete maternal antibody loss before 1 year old (Nguyen Tran et al., 2011, Yang et al., 2022). Particularly, models assuming no maternal immunity demonstrate significantly poorer fits to the data compared to those with maternal immunity, as shown in the obviously larger DIC value when assuming no maternal immunity (1,984 for model constructing FOI with piecewise function, 2,098 for model constructing FOI with Farrington’s function, see Supplementary Fig. 10 for DIC of models under different assumptions). This supports the presence of maternal immunity contained in the data sets.

## 3 Discussion

In this study, we applied Bayesian catalytic models jointly on case data and serological survey data collected in Ho Chi Minh City, Vietnam to infer age-time-specific transmission patterns of HFMD virus serotypes CV-A6, CV-A10, CV-A16, EV-A71 with consideration of maternal immunity. Our findings suggest the high-level transmission of CV-A6 and CV-A10, the susceptibility of older children aged 6–7 years and the strong association between severe illness and EV-A71, CV-A6 infections. In addition, based on the traditional catalytic model structure, we proposed a mathematical model framework describing maternal immunity in a feasible and adaptable manner, allowing for the incorporation of various assumptions of maternal immunity.

Our study contributes comprehensive estimations of HFMD dynamics, which encompass the viral impact on disease severity, serotype-varying transmission, time-heterogeneous spread pattern as well as the age-specific infection risk, and provides useful information to policy makers for effective interventions controlling the disease. By quantifying the difference between actual infections and hospitalised cases with scaling factors varied by serotypes, our estimations add statistical evidence about the differential impact of viral serotypes on disease severity. The comparable scaling factors of EV-A71 and CV-A6 indicate similar illness severity necessitating hospitalisation, as observed in the clinical data from other studies suggesting that CV-A6 and EV-A71 were the major serotypes causing severe cases (Li et al., 2016). Particularly, the clinical data provides detailed information about the specific clinical presentations related to various serotypes. EV-A71 infections have shown significant association with more severe symptoms such as vomiting, mental distress and lymphocyte abnormalities, which requires monitoring in emergence rooms or ICUs with higher fatality rate (Van Hoang et al., 2019, Thanh Nhan et al., 2020, Li et al., 2016). CV-A6 infections often present severe atypical HFMD symptoms such as higher fever, extended illness duration and wider-spread skin manifestations (Brooks David Kimmis et al., 2018, Aswathyraj et al., 2016, Broccolo et al., 2019). In contrast, CV-A16 is more likely to cause self-limited infections with mild symptoms (Mao et al., 2014) and CV-A10 infections are related with smaller proportion of skin lesions among hospitalised infections (Van Hoang et al., 2019, Thanh Nhan et al., 2020). Our study estimated the high-level transmission of CV-A6 and the silent transmission of CV-A10, which were masked by the disease severity in the distribution of hospitalised cases. The co-epidemic pattern of CV-A6 and CV-A10 also has been observed in Finland and China (Blomqvist et al., 2010, Xiao et al., 2019), with CV-A6 demonstrating high-level transmission documented in several countries including Italy, China, Singapore in the past few years (Broccolo et al., 2019, Di et al., 2014, Ang et al., 2015), while CV-A10 has been detected as one of the major transmitted serotypes in Wuhan, China (Yang et al., 2015). Furthermore, the cyclical pattern of HFMD viruses in Vietnam, which was observed in both the annual FOI and the hospitalised cases, was also observed in China (Wang et al., 2018, Le et al., 2022, Zhao et al., 2020), Malaysia (NikNadia et al., 2016) and Japan (Kanbayashi et al., 2017, Takahashi et al., 2018) with periodic period as 2–3 years.

Given the different characteristics of serotypes inferred from our research, the development of multivalent vaccines and active surveillance of different serotypes are needed to guide the implementation of strategies controlling HFMD spread in Vietnam despite the complexity of virological surveillance that enterovirus serotypes cause a wide range of symptoms including HFMD, herpangina etc. (Nguyet et al., 2020). Specifically, attention should be paid to CV-A6, which is an emerging major HFMD serotype recent years in terms of the large proportion of hospitalised cases, relative strong association with severe symptoms, high FOI and seroprevalence. The co-epidemic pattern of CV-A6 and CV-A10 may further increase the severity of CV-A6 with the rising risk of genomic recombination (Liu et al., 2014). Although CV-A10 exhibits a weaker association with severe illness, its high-level transmission indicates the possibility of causing outbreaks in the future. The low seroprevalence of CV-A16 and EV-A71 highlights the risk of future outbreaks in Ho Chi Minh City, Vietnam with cumulative population-level susceptibility till 2018 where a large EV-A71 outbreak has been observed after the low seroprevalence in 2017. Particularly, careful consideration should be given to the potential EV-A71 outbreaks regarding its serious harmfulness in causing severe illness.

HFMD has caused heavy economics burden in Vietnam for both the medical costs and nonmedical costs, especially for infections related to EV-A71 because of its association with severe illness (Thanh Nhan et al., 2019). Effective vaccine program is crucial in controlling the disease spread, reducing the economic burden of the healthcare system, and improving public health in Vietnam. Our inference of the HFMD age-structure transmission pattern provides useful insights into the implementation of vaccine programs in the region, especially the optimal vaccine age group and target vaccine age group to efficiently control the virus transmission and reduce the risk of severe illness. New-born infants were protected by maternal antibodies which were decreased to undetectable level by 1 year old (Nguyen Tran et al., 2011, Zhou et al., 2022, Fu et al., 2016, Yang et al., 2022). Children of age 1 to 2 years are estimated to be the most vulnerable to HFMD infections, given the highest risk of infection and the lowest cumulative immune protection, which was also observed in the case data of East and South China (Le et al., 2022, Di et al., 2014). The HFMD infection among children aged 1–2 years in Vietnam may be attributed to the household contacts and the contacts with peers of similar ages and childcare adults during attendance at day-care centres. Existing evidence underscores the crucial role of day-care centres and adults with asymptomatic infection in virus transmission among young children, as observed in the transmission of streptococcus pneumoniae in Vietnam (Toizumi et al., 2019, Thi Nguyen et al., 2019). Currently the HFMD vaccines target children aged 6 months to 5 years old or 2 months to 71 months, and recommend early vaccination before 12 months (Chinese Center for Disease Contro and Prevention, 2016, Nguyen et al., 2022). Our results support the guideline recommending early vaccination before the age of 1 year old, which enables children to acquire immune protection against the viruses prior to the period of highest infection risk. However, the estimated low seroprevalence of CV-A16 and EV-A71 signals the risk of potential outbreaks in the future even for older children aged >6 years, which has been observed in Thailand by a seroepidemiology study indicating a low seropositive rate in preschool children and a sharply increased seropositive rate in children >6 years old (Linsuwanon et al., 2014). To control the HFMD spread by vaccination, our study underscores the potential utility of covering wider age group than 6 months to 5 years old for the implementation of HFMD vaccine program in Vietnam. More research about the HFMD disease severity among children aged >5 years old, the vaccine protection duration and the corresponding health care burden is needed to assess the cost effectiveness of expanding the vaccine program to a broader population. Furthermore, the low seroprevalence for older age groups 6–7 years points out their susceptibility to CV-A16 and EV-A71, which should be taken into consideration within the vaccine program. Although this study is based on data sets collected in Ho Chi Minh City, the vaccination program in Vietnam should not be restricted to the city, which is a populous urban city and serves as the major centre of transportation in Vietnam. A nationwide vaccination program not only benefits children in other areas, but also plays an essential role in controlling the disease spread within Ho Chi Minh City.

Although our study provides reliable estimations of HFMD transmission patterns by considering maternal immunity and constructing more realistic model framework, we cannot make conclusive statements about maternal immunity without serological evidence from longitudinal studies. The serological survey data our model made inference on is a cross sectional data including only 2 subjects with age <1 year old. Our estimation that serotypes ranked from highest to lowest decreasing rate of maternal immunity are CV-A10, CV-A6, CV-A16, EV-A71 can be mainly explained by the age distribution of infections in case data. In the case data used in this study, the average age of patients infected by CV-A10 and CV-A6 is lower than that infected by CV-A16 and EV-A71, which indicates the absence of maternal immunity against CV-A10 and CV-A6 in an earlier age (Van Hoang et al., 2019). The simulation study indicates that the age distribution in case data is adequate in guiding the inference of maternal immunity while the inclusion of serological data reduces the estimation uncertainty and slightly improves the estimation accuracy even with the small sample size. However, the detailed change pattern of maternal immunity estimated in our model is conditional on the linear assumption or exponential assumption. The individual-level variation in serological status may also bias the results. Higher precision in the statistical estimations of maternal immunity might be achieved by releasing the change pattern assumptions and applying longitudinal cohort data with higher resolution.

Another main limitation of our study is that we do not consider cross reactivity between serotypes in our models. The animal models in preclinical studies about vaccine development (Zhang et al., 2018, Lim et al., 2018), the lack of seroconversion for heterotypic serotypes in a study collecting plasma samples from HFMD patients during and after the infection (Nguyet et al., 2020), the different infected virus serotypes among recurrent cases (Huang et al., 2018) and the lack of immune responses against other serotypes including CV-A6, CV-A10, CV-A16 among EV-A71 vaccinated children (Anasir and Poh, 2019) suggest the absence of cross reactivity. Additionally, the variation of sampling sites in the case data during 2018 that the sampling hospitals reduced from Children’s Hospital 1, Children’s Hospital 2 and Hospital for Tropical Diseases to Children’s Hospital 1 may induce bias to the data set and the results. The disease spread magnitude in 2018 may be underestimated due to the reduction of sampling sites. We investigated the impact of this limitation by multiplying a calibration rate to the number of cases in 2018 according to the hospital attendance data in previous studies (Anders et al., 2011, Thompson et al., 2015) (see Supplementary Discussion for details). The estimation of FOI with calibration presents a closely resembling dynamic pattern prior to 2018 as that without calibration while the FOI of serotypes CV-A6, CV-A10, EV-A71 in 2018 obviously increases after calibrating the case data (Supplementary Fig. 24). This suggests that the reduction of sampling sites primarily leads to the underestimation of virus transmission magnitude in 2018 with limited impact on the disease dynamic inference before 2018. Particularly, this sampling limitation is unlikely to have a substantial impact on the estimation of virus transmission patterns by age (Supplementary Fig. 24), assuming the similarity of age distributions among patients in 3 hospitals.

In the modelling perspective, we made joint use of case data and serological survey data and proposed a catalytic model framework with maternal immunity. As the gold standard of estimating virus transmission patterns, the serological survey data represents the disease spread patterns including asymptomatic infections with least bias. The case data in our study compensated for the sample size limitation of the serological data and reduced the uncertainty of the estimations. As evaluated in the simulation study, the model presents substantial competence of inferring the disease dynamic pattern where the joint usage of two data sets effectively enhances the model accuracy and stability. By applying the models with different assumptions, our study provided reliable estimations and explored the performance of various model assumptions. Without enough prior knowledge of maternal immunity, we simply applied exponential function, which is the function mathematically related to compartmental models and widely used in models for infectious diseases, and linear function to describe the diminishing pattern of maternal antibodies. By assuming exponential decline pattern of maternal immunity, the catalytic model with maternal immunity under our model framework is equivalent to the analytic solution of SIR model with maternal immunity (Pons-Salort et al., 2023, Hens et al., 2012). The exponential function estimates a decreasing rate at around 1 to 3 (Supplementary Table 4) while a serological study about enterovirus D68 in England estimates a maternal antibody decreasing rate at around 0.2 to 2 (Pons-Salort et al., 2023). The estimation discrepancy may be attributed to the region diversity and serotype difference. Interestingly, the linear function exhibits greater robustness across various proportions of children born with maternal immunity and better fitting between model estimations and data sets as shown in the smaller DIC. This underscores the potential efficacy of the linear function in models for infectious diseases.

## 4 Conclusion

In conclusion, our results provide statistical evidence supporting the strong association between severe illness and infections by EV-A71, CV-A6. The models identify the high-level transmission of CV-A6 and CV-A10 which is not directly revealed in the distribution of hospitalised cases. The low seroprevalence of CV-A16, EV-A71 emphasises the risk of future outbreaks and the potential necessary of vaccinating children with older age than the current vaccine guideline. Collectively, our results highlight the importance of active surveillance for different serotypes and the essentiality of developing multivalent vaccines, offer useful insights for the establishment of HFMD vaccine program in Vietnam.

## 5 Materials and methods

### 5.1 Data collection

The serological survey data in this study was collected from a serial seroepidemiology study conducted by Oxford University Clinical Research Unit (OUCRU), Vietnam from 2009 (Lam et al., 2019, Quan et al., 2018, Duy Nhat et al., 2017). This serial seroepidemiology study built a serum bank including residual serum samples collected from inpatients and outpatients of 10 hospitals located in 10 provinces of Vietnam. The recruitment of subjects was not related to their diagnosis of disease or their reasons of visiting the hospital. In our study, the serological survey data includes 48 residual serum samples collected in 2015 and 52 residual serum samples collected in 2017. The residual serum samples in the data set were randomly selected from the subjects with age <7 years in this seroepidemiology study at the Hospital for Tropical Diseases in Ho Chi Minh City. The neutralising antibody titre of residual serum samples against HFMD virus serotypes CV-A6, CV-A10, CV-A16, EV-A71 was tested by neutralisation assay. Details of the assay are given in Nguyet et al. (2020). In brief, the number of dilutions tested was from 1:8 to 1:512. Each dilution was tested in quadruplicate. The viral strain used to measure neutralisation for CV-A10, CV-A16, EV-A71 was isolated from HFMD patients enrolled in the clinical study (Van Hoang et al., 2019). The viral strain for CV-A6 was isolated from the virus archive of Pasteur Institute in Ho Chi Minh City. To align with established standards, we adopted a seropositive threshold of 1:8 for neutralising antibody titres, consistent with the protocol utilised in Nguyet et al. (2020) and widely applied in other serological studies (Zhou et al., 2022).

The case data analysed in this study was collected at Children’s Hospital 1, Children’s Hospital 2, and Hospital for Tropical Diseases in Ho Chi Minh City, Vietnam from 2013 to 2018. HFMD patients with age smaller than 12 years old were recruited in the study. Written informed consent was given by the guardian of the participants. Rectal and throat swabs were collected at enrolment. Infecting virus serotypes were determined by a multiplex real-time RT-PCR on throat swabs, while rectal swabs were used if the PCR test result of throat swabs was negative (Thanh et al., 2015). Basic demographics information and clinical characteristics data were collected as well (Van Hoang et al., 2019, Thanh Nhan et al., 2020). Because of the resource limitation, the recruitment of patients was only conducted at Children’s Hospital 1 in 2018. The case data includes 1,772 cases infected by serotypes CV-A6, CV-A10, CV-A16, EV-A71 and 985 cases infected by other virus serotypes or with negative results. Based on this data set, the clinical presentation of different infected serotypes, frequency summary of patients with different symptoms and infected serotypes, phenotypic analysis of EV-A71 subgenogroups, time-varying distribution of the cases were analysed and reported previously (Van Hoang et al., 2019, Thanh Nhan et al., 2020). In this study, we focus on the models inferring age-time-specific infection risk, i.e., FOI for 4 virus serotypes CV-A6, CV-A10, CV-A16, EV-A71 and provide valuable insights to policymakers based on our findings.

### 5.2 Catalytic model framework with maternal immunity

We applied catalytic model as the basic model structure to estimate FOI, which is defined as the rate that the susceptible individuals acquire infections in a population per unit time (Muench, 2013). The catalytic model assumes life-long immunity acquired from natural infection, which means that each individual can be infected once and remain seropositive thereafter. In this section, we will present a modified catalytic model framework with adaptable structure of maternal immunity. All details about the models are in Supplementary Methods. For simplicity, we present the modified catalytic model framework assuming constant FOI and age-varying spread pattern of the disease. Let λ denote the constant FOI, g(a) denote the proportion of people aged a losing maternal immunity among those born with maternal immunity, $s{\left(a\right)}_{\text{MI}}$ denote the proportion of people aged a susceptible to the virus among those born with maternal immunity, $s{\left(a\right)}_{\text{no MI}}$ denote the susceptible proportion of people aged a among those born without maternal immunity. Following the general catalytic model framework with no maternal immunity, $s{\left(a\right)}_{\text{no MI}}=e^{-\lambda a}$ (Muench, 2013). In our model framework with maternal immunity, we derived that

(1) $$s{\left(a\right)}_{\text{MI}}=\int_{0}^{a}e^{-\left(a-x\right)\lambda }\frac{dg\left(x\right)}{dx}dx,$$

where g(a) is a monotonically increasing, differentiable function restricted between 0 and 1. Model (1) can be interpreted as that the probability of being susceptible to the virus at age a is $e^{-\left(a-x\right)\lambda }$ if the individual loses maternal immunity at age x<a. In other words, one individual becomes susceptible to the disease because of losing maternal immunity and lacking immune protection obtained from natural infection. By using $g\left(a\right)=1-e^{-\eta a}$, $s{\left(a\right)}_{\text{MI}}$ in Model (1) is homogeneous to that in SIR model with maternal immunity where η is the rate of losing maternal immunity per unit time (Pons-Salort et al., 2023, Hens et al., 2012). In a population with a subset of children born with maternal immunity, the proportion of people aged a susceptible to the virus is calculated as

(2) $$s\left(a\right)=q\times s{\left(a\right)}_{\text{MI}}+\left(1-q\right)\times s{\left(a\right)}_{\text{no MI}},$$

where q is the proportion of children born with maternal immunity.

### 5.3 Maternal immunity description and Force of Infection estimation

In this study, we applied two mathematical descriptions of maternal immunity and two constructions of age-time-specific FOI under the modified catalytic model framework Eq. (1). For maternal immunity, we assumed that all new-born infants were protected by maternal immunity, and maternal immunity gradually diminished and completely disappeared before age = 1 year old (Nguyen Tran et al., 2011, Zhou et al., 2022, Fu et al., 2016, Yang et al., 2022). Sensitivity test was conducted with different proportion of infants born with maternal immunity. We applied exponential functions $g_{\text{exp}}^{S}\left(a\right)$ and linear functions $g_{\text{lin}}^{S}\left(a\right)$ to describe the change pattern of maternal immunity by age a for virus serotypes S respectively as follows.

(3) $$g_{\text{exp}}^{S}\left(a\right)≔1-e^{-\eta^{S}a},\;\;\eta^{S}>0;$$

$$g_{\text{lin}}^{S}\left(a\right)≔\min\left\{\xi^{S}a,1\right\},\;\;\xi^{S}\geq 1.$$

Let $\lambda_{A,t}^{S}$ denote the average FOI with 1-year band of age group A, time group t and virus serotype S. We estimated $\lambda_{A,t}^{S}$ with A∈{[0,1),[1,2),…,[6,7),[7,11]}, t∈{2004−2011,2012,2013,…,2018}, S∈{CV-A6,CV-A10,CV-A16,EV-A71}. We applied two formulations of FOI with piecewise function $\lambda_{\text{Pw},A,t}^{S}$ and Farrington’s function $\lambda_{\text{Ft},A,t}^{S}$ respectively as below.

(4) $$\lambda_{\text{Pw},A,t}^{S}=\gamma_{t}^{S}\times \gamma_{A},\;\;\gamma_{t}^{S},\gamma_{A}\geq 0;$$

$$\lambda_{\text{Ft},A,t}^{S}=\int_{a}^{a+1}\beta_{t}^{S}xe^{-\beta_{2}x}dx\;\;\text{where}A=\left[a,a+1\right).$$

The piecewise function uses $\gamma_{t}^{S}$ to quantify the transmission ability of the virus by time and virus serotypes and $\gamma_{A}$ to quantify the impact of age-specific contact pattern on FOI. The Farrington’s function applies the function $\lambda_{a,t}^{S}=\beta_{t}^{S}ae^{-\beta_{2}a}$ as a smooth mathematical description of FOI, gives a stronger assumption to the change pattern of FOI that FOI increases by age, reaches a peak and decreases (Paddy Farrington, 1990). (See Supplementary Equation 1 for calculation details of $\lambda_{\text{Ft},A,t}^{S}$.)

### 5.4 Risk of infection, susceptible proportion and seroprevalence

The risk of infection $\text{Pr}{\left(\text{infection}\right)}_{A,t}^{S}$, which is mathematically defined as the probability of being infected by virus serotype S during calendar year t for the population subgroup who is in the 1-year band age group A and susceptible to the disease virus, is calculated as (Muench, 2013)

(5) $$\text{Pr}{\left(\text{infection}\right)}_{A,t}^{S}=1-e^{-\lambda_{A,t}^{S}}.$$

Susceptible proportion (denoted as $\text{Pr}{\left(\text{susceptible}\right)}_{A,t}^{S}$) is mathematically defined as the probability of being susceptible to virus serotype S before and during time period t for individuals in age group A. It is calculated with Eq. (2) using the corresponding maternal immunity mathematical description (Supplementary Equation 2,3). Seroprevalence is defined as the proportion of people with immune protection against the virus. It can be calculated as 1 minus the corresponding susceptible proportion.

### 5.5 Model fitting and model evaluation

The number of reported cases in case data is modelled by Poisson distribution with the expected number of reported cases $E_{A,t}^{S}$ calculated as

(6) $$E_{A,t}^{S}=N_{A,t}\times \text{Pr}{\left(\text{susceptible}\right)}_{A-1,t-1}^{S}\times \text{Pr}{\left(\text{infection}\right)}_{A,t}^{S}\times \phi^{S},$$

where $N_{A,t}$ is the population size in Ho Chi Minh City, Vietnam for age group A during calendar year t, $\phi^{S}$ is the scaling factor of virus serotype S accounting for the difference between infections and reported cases induced by asymptomatic infections, minor infections, or some unrecorded infections due to data collection technique problems (Rodriguez-Barraquer et al., 2019). As the data collection procedures were homogenous for age groups and calendar years, the difference between the scaling factor $\phi^{S}$ can be attributed to the variation of disease severity across serotypes. The log likelihood of individual i with seronegative result ($l^{S_{i},\text{neg}}$) and seropositive result ($l^{S_{i},\text{pos}}$) against virus serotype $S_{i}$ in the serological survey data is as follows (Muench, 2013).

(7) $$l^{S_{i},\text{neg}}=\log\left(\text{Pr}{\left(\text{susceptible}\right)}_{A_{i},t_{i}}^{S_{i}}\right);$$

$$l^{S_{i},\text{pos}}=\log\left(1-\text{Pr}{\left(\text{susceptible}\right)}_{A_{i},t_{i}}^{S_{i}}\right).$$

Though the case data was collected from 2013 to 2018 and the serological survey data was collected in 2015 and 2017, FOI from 2004 to 2012 can be estimated as in Fig. 2 because subjects in the data sets provide information of FOI during their lifetime. By taking the median 1-year-band age group [9,10) of the older age group [7,11] to fit the models, we assumed time-homogeneous FOI from 2004 to 2011 because of the limited information included in the data where there were no corresponding cases or residual serum samples collected during 2004 to 2011.

The models were implemented under Bayesian model structure with hierarchical priors for parameters constructing FOI. For the age group 0–1 year old, the models were applied on the case number with month unit and the residual serum samples with exact age to estimate the maternal immunity. Details of the prior distributions are in Supplementary Equation 4–8. All the prior distributions are uninformative in shaping the posterior distributions, which were checked by comparing the density of prior distributions and posterior distributions (Supplementary Fig. 25–28). The population size data of Ho Chi Minh City, Vietnam was obtained from Vietnam General Statistics Office (Vietnam General Statistics Office, 2020) and United Nations Database (United Nations Statistics Division, 2022). The Bayesian models were fitted by generating HMC samples with rstan (Version 2.18.2) (Carpenter et al., 2017). The posterior distributions of the parameters were estimated by 4 HMC chains with 10,000 iterations each chain, of which the first 1,000 iterations were warm-up samples and removed as burn-in. The convergence of the 4 chains was checked by the minimum effective sample size, the maximum R hat value, and the trace plots of the parameters with top 4 smallest effective sample size. The model performance was visualised by comparing the data and the estimated values, and assessed by DIC which was widely used in evaluating model fitting effect of Bayesian models (Spiegelhalter et al., 2002).

### 5.6 Simulation study

We simulated 100 case data sets, 100 serological data sets with the same sample size as the data used in this study, 100 serological data sets with 10 samples per age group per year and 100 serological data sets with 15 samples per age group per year. The model was implemented on the 100 simulated data sets, using the case data alone as well as in combination with the serological survey data. The performance of the model at inferring FOI, the maternal immunity decline pattern and the serotype-specific scaling factor was measured using the average estimate, estimate range and coverage rate across the 100 data sets. Specifically, the coverage rate is calculated as the proportion of simulations that the pre-defined parameter value falls within the 95% confidence interval among the 100 simulations.

## CRediT authorship contribution statement

**Yining Chen:** Writing – review & editing, Writing – original draft, Visualization, Validation, Software, Methodology, Funding acquisition, Formal analysis, Conceptualization. **Lam Anh Nguyet:** Data curation. **Le Nguyen Thanh Nhan:** Investigation, Data curation. **Phan Tu Qui:** Resources. **Le Nguyen Truc Nhu:** Data curation. **Nguyen Thi Thu Hong:** Data curation. **Nguyen Thi Han Ny:** Data curation. **Nguyen To Anh:** Investigation, Data curation. **Le Kim Thanh:** Investigation. **Huynh Thi Phuong:** Resources, Data curation. **Nguyen Ha Thao Vy:** Resources, Data curation. **Nguyen Thi Le Thanh:** Investigation. **Truong Huu Khanh:** Resources. **Nguyen Thanh Hung:** Investigation. **Do Chau Viet:** Investigation. **Nguyen Tran Nam:** Investigation. **Nguyen Van Vinh Chau:** Resources, Investigation. **H. Rogier van Doorn:** Supervision, Resources. **Le Van Tan:** Writing – review & editing, Validation, Supervision, Resources, Project administration, Investigation, Funding acquisition. **Hannah Clapham:** Writing – review & editing, Validation, Supervision, Project administration, Methodology, Funding acquisition, Conceptualization.

## Declaration of competing interest

The authors declare that they have no known competing financial interests or personal relationships that could have appeared to influence the work reported in this paper.

## Data Availability

The original data, preprocessed data, model results and the corresponding codes are available at https://github.com/chenyn226/hfmd-foi.git.

## Appendix A Supplementary data

MMC S1 Supplementary material: catalytic model with maternal immunity for HFMD in Vietnam.
