# Supplementary material for: Age-time-specific transmission of hand-foot-and-mouth disease enterovirus serotypes in Vietnam: A catalytic model with maternal immunity
Source: Epidemics. 2024 Mar;46:100754. doi: 10.1016/j.epidem.2024.100754 (PMC10945305; doi:10.1016/j.epidem.2024.100754)
Supplement: MMC S1 — Supplementary material: catalytic model with maternal immunity for HFMD in Vietnam. [file mmc1.pdf]

Supplementary Materials: Age-time-specific transmission of hand-foot-and-mouth disease enterovirus serotypes in Vietnam: a catalytic model with maternal immunity

Results

Model fitting evaluation

Table 1: Sample size of serological survey data by time and age

Age group (years)	Number of residual serum samples	
	2015 ($N = 48$)	2017 ($N = 52$)
$0 - 1$	2	0
$1 - 2$	10	3
$2 - 3$	12	10
$3 - 4$	11	11
$4 - 5$	5	9
$5 - 6$	4	9
$6 - 7$	4	10

Table 2: Minimum effective sample size and maximum R hat value for models.

	Model *LinPw*	Model *LinFt*	Model *ExpPw*	Model *ExpFt*
minimum effective sample size	5,428	4,418	4,930	3,850
maximum R hat value	1.0007	1.0009	1.0007	1.0007

The number of effective parameters, which was calculated as the log likelihood with posterior mean of parameters subtracting the average log likelihood over the posterior distribution and multiple by 2, is positive and reasonable for models *LinPw* (42.27), *LinFt* (35.62), *ExpPw* (43.60), *ExpFt* (37.71) as the total number of parameters is 51 in models *LinPw*, *ExpPw*, and 43 in models *LinFt*, *ExpFt*.

The trace plots of 4 parameters with top 4 smallest effective sample size are Figure 1, Figure 2, Figure 3, Figure 4. The estimated case number of 4 models is in Figure 5. The estimated seroprevalence of 4 models is in Figure 6.

Force of Infection and HFMD disease severity estimates

The estimations about disease severity of infections by different serotypes are as follows. All 4 models estimate that CV-A10 has the smallest scaling factor (0.0007, 95% confidence interval (CI): 0.0006 – 0.0009). By taking the scaling factor of CV-A10 as the reference, the relative value of the scaling factor of virus serotypes CV-A6, CV-A16, EV-A71 is in Table 3.

The estimation of FOI in 4 models is in Figure 7.

Table 3: Relative scaling factor (estimate, 95% CI) of virus serotypes CV-A6, CV-A16, EV-A71 with CV-A10 as reference

	CV-A6	CV-A16	EV-A71
Model *LinPw*	3.17 (2.35 – 4.31)	2.32 (1.60 – 3.46)	4.31 (2.97 – 6.60)
Model *LinFt*	3.21 (2.38 – 4.39)	2.35 (1.61 – 3.52)	4.40 (2.99 – 6.72)
Model *ExpPw*	3.12 (2.27 – 4.34)	1.70 (1.12 – 2.65)	3.24 (2.18 – 4.99)
Model *ExpFt*	3.14 (2.26 – 4.42)	1.61 (1.07 – 2.53)	3.03 (2.08 – 4.61)

Maternal immunity insights from modeling

Table 4: Estimation (estimate, 95% CI) of the age (years) that all children lose maternal immunity in Model *LinPw*, *LinFt* ($1/\xi^S$) and estimation (estimate, 95% CI) of the maternal immunity decreasing rate in Model *ExpPw*, *ExpFt* (η^S).

	CV-A6	CV-A10	CV-A16	EV-A71
	The age (years) that all children lose maternal immunity			
Model *LinPw*	0.89 (0.76 – 0.98)	0.69 (0.48 – 0.91)	0.95 (0.83 – 0.99)	0.96 (0.85 – 0.99)
Model *LinFt*	0.93 (0.82 – 0.99)	0.77 (0.54 – 0.95)	0.97 (0.86 – 0.99)	0.98 (0.89 – 0.99)
	The maternal immunity decreasing rate			
Model *ExpPw*	2.20 (1.91 – 2.64)	3.19 (2.52 – 4.29)	1.35 (1.08 – 1.80)	1.23 (0.98 – 1.65)
Model *ExpFt*	2.14 (1.88 – 2.50)	3.08 (2.47 – 4.07)	1.30 (1.08 – 1.68)	1.14 (0.96 – 1.43)

Simulation study and sensitivity test

Table 5: The simulation value, average estimate, range of estimates and coverage rate for parameters describing maternal immunity decline pattern and scaling factors in simulation study.

Parameters	Simulation value	Models using only case data		Models using case data and serological data with the same sample size as used in the study		Models using case data and serological data with 10 samples per age-time group		Models using case data and serological data with 15 samples per age-time group	
		Average estimates (range)	Coverage rate	Average estimates (range)	Coverage rate	Average estimates (range)	Coverage rate	Average estimates (range)	Coverage rate
**The age that all children lose maternal immunity against the corresponding serotype**									
CV-A6	0.67	0.62 (0.38,0.88)	94%	0.64 (0.42,0.91)	95%	0.64 (0.45,0.90)	95%	0.64 (0.38,0.90)	96%
CV-A10	0.50	0.49 (0.28,0.84)	96%	0.50 (0.29,0.73)	98%	0.50 (0.34,0.72)	98%	0.50 (0.35,0.74)	97%
CV-A16	0.75	0.72 (0.48,0.93)	99%	0.73 (0.50,0.93)	99%	0.73 (0.50,0.92)	99%	0.73 (0.50,0.90)	97%
EV-A71	0.83	0.84 (0.63,0.94)	100%	0.83 (0.63,0.94)	100%	0.84 (0.62,0.94)	99%	0.84 (0.67,0.94)	100%
**Serotype-specific scaling factor**									
CV-A6	0.0022	0.0029 (0.0019,0.0057)	81%	0.0024 (0.0020,0.0029)	92%	0.0024 (0.0019,0.0031)	89%	0.0024 (0.0019,0.0033)	89%
CV-A10	0.0007	0.0009 (0.0005,0.0017)	82%	0.0008 (0.0006,0.0010)	80%	0.0008 (0.0005,0.0010)	85%	0.0008 (0.0006,0.0012)	86%
CV-A16	0.0016	0.0019 (0.0009,0.0033)	99%	0.0016 (0.0011,0.0023)	96%	0.0016 (0.0011,0.0029)	95%	0.0016 (0.0012,0.0025)	96%
EV-A71	0.0030	0.0039 (0.0018,0.0064)	100%	0.0030 (0.0020,0.0045)	97%	0.0030 (0.0022,0.0048)	97%	0.0030 (0.0021,0.0039)	96%

Table 6: Effective number of parameters for the calculation of DIC

Proportion of children born with maternal immunity	Model *LinPw*	Model *ExpPw*	Model *LinFt*	Model *ExpFt*
50%	41.79	42.90	35.58	37.47
60%	41.86	42.85	35.53	37.58
70%	41.88	43.30	35.77	37.65
80%	42.29	43.28	35.99	37.60
90%	42.27	43.39	35.82	37.77
no maternal immunity	41.61		36.10	

The DIC for models with different proportion of children born with maternal immunity is in Figure 10. The estimation of case number, seroprevalence and FOI in Model *LinPw* with different proportion of children born with maternal immunity is in Figure 11, Figure 12, Figure 13. The estimation of case number, seroprevalence and FOI in Model *LinFt* with different proportion of children born with maternal immunity is in Figure 14, Figure 15, Figure 16. The estimation of case number, seroprevalence and FOI in Model *ExpPw* with different proportion of children born with maternal immunity is in Figure 17, Figure 18, Figure 19. The estimation of case number, seroprevalence and FOI in Model *ExpFt* with different proportion of children born

with maternal immunity is in Figure 20, Figure 21, Figure 22. The estimation of the proportion of children losing maternal immunity during the first year life time is in Figure 23.

Discussion

In the case data, the sampling hospitals reduced from Children's Hospital 1 (CH1), Children's Hospital 2 (CH2) and Hospital for Tropical Diseases (HTD) to CH1 in 2018. We investigated the impact of this limitation by multiplying a calibration rate to the number of cases in 2018 according to the hospital attendance data in previous studies. In the study *Epidemiological factors associated with dengue shock syndrome and mortality in hospitalized dengue patients in Ho Chi Minh City, Vietnam* (doi: 10.4269/ajtmh.2011.10-0476.), the admission size of HTD, CH1, CH2 is 18,980, 33,542 and 225,35. The number of HFMD cases in 3 hospitals could be approximated by 2.24 times the cases in CH1 where the calibration rate 2.24 is calculated as the total admission size of 3 hospitals divided by that of CH1. In study *The impact of environmental and climatic variation on the spatiotemporal trends of hospitalized pediatric diarrhea in Ho Chi Minh City, Vietnam* (doi: 10.1016/j.healthplace.2015.08.001.), there were 443,295 children under 16 years old admitted to CH1 and CH2 from 2005 to 2010 and 36,624 children admitted to HTD from 2008 to 2010. By using the total admission size per year in 3 hospitals divided by that of CH1, the calibration rate is calculated as 2.33. The similar values of calibration rate calculated based on two studies support its validity. We used the calibration rate averaging over the two values calculated from two studies, i.e., 2.29.

The estimation of FOI in Model *LinPw* based on case data with and without calibration is in Figure 24.

Methods

Maternal immunity description and Force of Infection estimation

$\lambda^S_{\mathrm{Ft},A,t}$ is modified from Farrington's function. Let $\lambda^S_{a,t}$ denote the smooth function of FOI for age a, time group t and virus serotype S. We modified the construction of FOI in Farrington's function and assumed that

$$\lambda^S_{a,t} = \beta^S_t a e^{-\beta_2 a}, \;\; \beta^S_t, \beta_2 \geq 0,$$

where $\lambda^S_{a,t}$ reaches the peak at $a = 1/\beta_2$, β^S_t controls the peak value, increasing and decreasing pattern of $\lambda^S_{a,t}$ before and after reaching the peak. The homogeneity of β_2 for different t and S implies the assumption that the age group with largest risk of infection is the same for different year groups and virus serotypes. The average FOI $\lambda^S_{\mathrm{Ft},A,t}$ can be calculated as

$$\begin{aligned}\lambda^S_{\mathrm{Ft},A,t} &= \int_a^{a+1} \beta^S_t x e^{-\beta_2 x} dx \;\text{ where } A = [a, a+1) \\ &= \frac{\beta^S_t}{\beta_2} e^{-\beta_2 a} \left[(a + \frac{1}{\beta_2})(1 - e^{-\beta_2}) - e^{-\beta_2} \right].\end{aligned} \tag{1}$$

Risk of infection, susceptible proportion and seroprevalence

If we assume that all children born with maternal immunity, $s(a) = s(a)_{\mathrm{MI}}$. The susceptible proportion $\Pr(\text{susceptible})^S_{A,t}$, which is mathemetically defined as the probability of being susceptible to virus serotype S before and during time period t for individuals in age group A, is calculated with the modified catalytic model below

$$s(a) = \int_0^a e^{-(a-x)\lambda} \frac{dg(x)}{dx} dx,$$

where $s(a)$ is the proportion of people aged a susceptible to the virus, $g(x)$ is the proportion of people aged x losing maternal immunity. By assuming exponential functions $g^S_{\text{exp}}(x) = 1 - e^{-\eta^S x}$ and linear functions $g^S_{\text{lin}}(x) = \min\{\xi^S x, 1\}$ for the proportion of people aged x losing maternal immunity, susceptible proportion can be calculated as

$$\begin{aligned}
\Pr(\text{susceptible})^S_{\text{exp},A,t} &= \frac{\eta^S}{\eta^S - \lambda^S_{[0,1),t}} e^{-\sum_{(k,t_k)\in\{A,t\}} \lambda^S_{[k,k+1),t_k}}; \\
\Pr(\text{susceptible})^S_{\text{lin},A,t} &= \frac{\xi^S}{\lambda^S_{[0,1),t}} \left(e^{\frac{\lambda^S_{[0,1),t}}{\xi^S}} - 1 \right) e^{-\sum_{(k,t_k)\in\{A,t\}} \lambda^S_{[k,k+1),t_k}},
\end{aligned} \tag{2}$$

where $\{A,t\} = \{(0,t-a), \cdots, (a-2,t-2), (a-1,t-1), (a,t)\}$ for $A = [a, a+1)$, subscript exp and lin refers to the mathematical form of susceptible proportion with maternal immunity constructed by exponential functions and linear functions.

The calculation details of $\Pr(\text{susceptible})^S_{\text{exp},A,t}$ is as follows. For age $a < 1$,

$$\begin{aligned}
\Pr(\text{susceptible})^S_{\text{exp},a,t} &= \int_0^a e^{-(a-x)\lambda^S_{[0,1),t}} \frac{d(1-e^{-\eta^S x})}{dx} dx \\
&= \frac{\eta^S}{\eta^S - \lambda^S_{[0,1),t}} (e^{-\lambda^S_{[0,1),t} a} - e^{-\eta^S a}).
\end{aligned}$$

For age $a = 1$, based on the assumption that maternal immunity completely disappears before age 1 year old, $g^S_{\text{exp}}(1) \approx 1$, i.e. $e^{-\eta^S} \approx 0$. Then $\Pr(\text{susceptible})^S_{\text{exp},1,t}$ can be simplified as

$$\Pr(\text{susceptible})^S_{\text{exp},1,t} = \frac{\eta^S}{\eta^S - \lambda^S_{[0,1),t}} e^{-\lambda^S_{[0,1),t}}.$$

For age groups $A = [a, a+1), a \in \mathbb{N}$,

$$\Pr(\text{susceptible})^S_{\text{exp},A,t} = \frac{\eta^S}{\eta^S - \lambda^S_{[0,1),t}} e^{-\sum_{(k,t_k)\in\{A,t\}} \lambda^S_{[k,k+1),t_k}}.$$

The calculation details of $\Pr(\text{susceptible})^S_{\text{lin},A,t}$ is as follows. For age $a \le 1/\xi^S$,

$$\begin{aligned}
\Pr(\text{susceptible})^S_{\text{lin},a,t} &= \int_0^a e^{-(a-x)\lambda^S_{[0,1),t}} \frac{d(\xi^S x)}{dx} dx \\
&= \frac{\xi^S}{\lambda^S_{[0,1),t}} e^{-\lambda^S_{[0,1),t} a} (e^{\lambda^S_{[0,1),t} a} - 1).
\end{aligned}$$

For age $a \in (1/\xi^S, 1)$, $g^S_{\text{lin}}(a) = 1$,

$$\begin{aligned}
\Pr(\text{susceptible})^S_{\text{lin},a,t} &= \Pr(\text{susceptible})^S_{\text{lin},1/\xi^S,t} \times e^{-\lambda^S_{[0,1),t}(a - \frac{1}{\xi^S})} \\
&= \frac{\xi^S}{\lambda^S_{[0,1),t}} e^{-\lambda^S_{[0,1),t} \frac{1}{\xi^S}} (e^{\lambda^S_{[0,1),t} \frac{1}{\xi^S}} - 1) e^{-\lambda^S_{[0,1),t}(a - \frac{1}{\xi^S})} \\
&= \frac{\xi^S}{\lambda^S_{[0,1),t}} e^{-\lambda^S_{[0,1),t} a} (e^{\frac{\lambda^S_{[0,1),t}}{\xi^S}} - 1).
\end{aligned}$$

Combining the equations above, for age $a < 1$, $\Pr(\text{susceptible})^S_{\text{lin},a,t}$ can be calculated as

$$\Pr(\text{susceptible})^S_{\text{lin},a,t} = \frac{\xi^S}{\lambda^S_{[0,1),t}} e^{-\lambda^S_{[0,1),t} a} (e^{\lambda^S_{[0,1),t} \min\{a, 1/\xi^S\}} - 1).$$

For age $a=1$, $\Pr(\text{susceptible})^S_{\text{lin},1,t}$ can be calculated as

$$\Pr(\text{susceptible})^S_{\text{lin},1,t} = \frac{\xi^S}{\lambda^S_{[0,1),t}} e^{-\lambda^S_{[0,1),t}} (e^{\frac{\lambda^S_{[0,1),t}}{\xi^S}} - 1).$$

For age groups $A = [a, a+1), a \in \mathbb{N}$,

$$\Pr(\text{susceptible})^S_{\text{lin},A,t} = \frac{\xi^S}{\lambda^S_{[0,1),t}} \left(e^{\frac{\lambda^S_{[0,1),t}}{\xi^S}} - 1 \right) e^{-\sum_{(k,t_k)\in\{A,t\}} \lambda^S_{[k,k+1),t_k}}.$$

Sensitivity test

In sensitivity test, we assumed the proportion of children born with maternal immunity is q, $q = \{50\%, 60\%, \cdots, 90\%\}$. The catalytic model with maternal immunity is

$$s(a) = q \times \int_0^a e^{-(a-x)\lambda} \frac{dg(x)}{dx} dx + (1-q)e^{-a\lambda}.$$

The susceptible proportion $\Pr(\text{susceptible})^S_{A,t}$ is calculated as

$$\begin{aligned} \Pr(\text{susceptible})^S_{\exp,A,t} &= \left[\frac{q\eta^S}{\eta^S - \lambda^S_{[0,1),t}} + (1-q) \right] e^{-\sum_{(k,t_k)\in\{A,t\}} \lambda^S_{[k,k+1),t_k}}; \\ \Pr(\text{susceptible})^S_{\text{lin},A,t} &= \left[\frac{q\xi^S}{\lambda^S_{[0,1),t}} \left(e^{\frac{\lambda^S_{[0,1),t}}{\xi^S}} - 1 \right) + (1-q) \right] e^{-\sum_{(k,t_k)\in\{A,t\}} \lambda^S_{[k,k+1),t_k}}. \end{aligned} \tag{3}$$

Model fitting and model evaluation

The models were implemented under Bayesian model structure with hierarchical priors for parameters constructing FOI. The notation $\text{Normal}(\mu, \sigma^2)$ refers to normal distribution with mean μ and standard deviance σ; The notation $\text{HalfCauchy}(\mu, \sigma)$ refers to half Cauchy distribution with location μ and scale σ; The notation $\text{Exponential}(\theta)$ refers to exponential distribution with mean $1/\theta$; The notation $\text{Gamma}(\alpha, \beta)$ refers to Gamma distribution with shape α and rate β. The Bayesian hierarchical priors of parameters in $\lambda^S_{\text{Pw},A,t}$ are as follows:

$$\begin{aligned} &\log(\gamma^S_t) \sim \text{Normal}(\gamma_0, \sigma^2_{\gamma_1}), \gamma_0 \sim \text{Normal}(0, 5^2), \sigma_{\gamma_1} \sim \text{HalfCauchy}(0,1); \\ &\log(\gamma_A) \sim \text{Normal}(0, \sigma^2_{\gamma_2}), \sigma_{\gamma_2} \sim \text{HalfCauchy}(0,1). \end{aligned} \tag{4}$$

The Bayesian hierarchical priors of parameters in $\lambda^S_{\text{Ft},A,t}$ are as follows:

$$\begin{aligned} &\log(\beta^S_t) \sim \text{Normal}(\beta_0, \sigma^2_\beta), \beta_0 \sim \text{Normal}(0, 5^2), \sigma_\beta \sim \text{HalfCauchy}(0,1); \\ &\beta_2 \sim \text{Exponential}(1). \end{aligned} \tag{5}$$

The prior for η^S in $g^S_{\exp}$ is

$$\eta^S \sim \text{Gamma}(4, 2). \tag{6}$$

The prior for ξ^S in g^S_{lin} is

$$\xi^{S*} \sim \text{Exponential}(1), \text{ where } \xi^S = \xi^{S*} + 1. \tag{7}$$

The choices of priors for η^S and ξ^S have the same mean and variance, which indicates similar prior knowledge linked to the models assuming maternal immunity with exponential functions and linear functions. The scaling factor ϕ^S has prior distribution as

$$\log(\frac{1}{\phi^S}) \sim \text{Exponential}(0.1). \tag{8}$$

The priors are checked by comparing the density plots of prior distributions and posterior distributions as in Figure 25, Figure 26, Figure 27, Figure 28. According the figures, the prior distributions are relatively flat compared to the posterior distributions, which indicates that prior distributions are uninformative in shaping the posterior distributions.

Figures

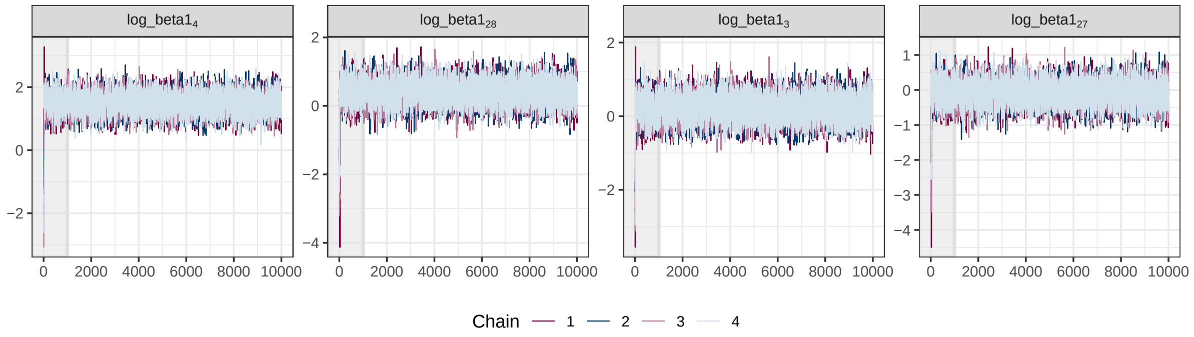

Figure 1: Trace plot of Model *LinPw*

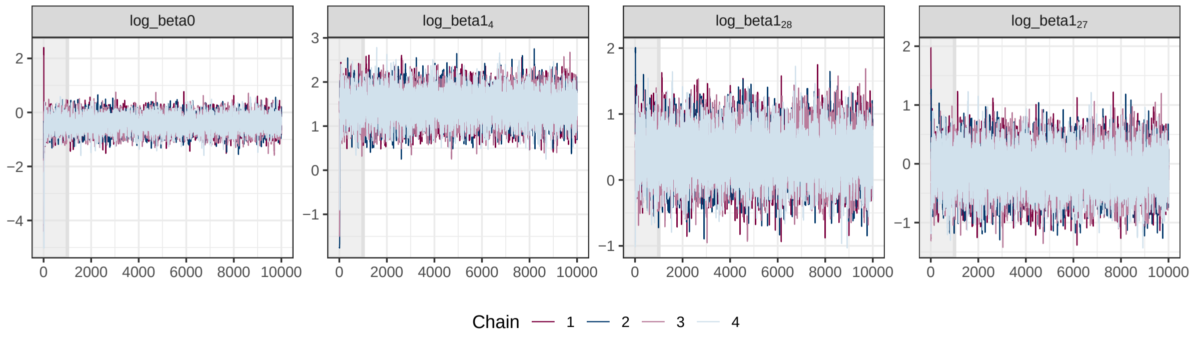

Figure 2: Trace plot of Model *LinFt*

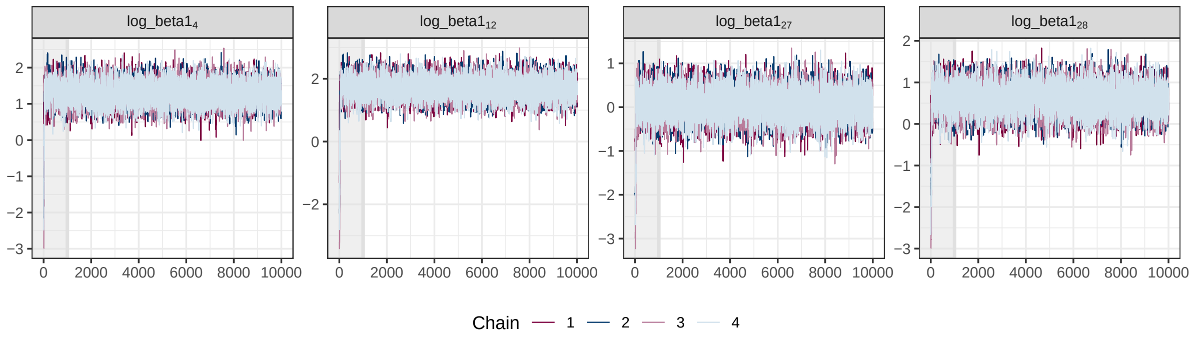

Figure 3: Trace plot of Model *ExpPw*

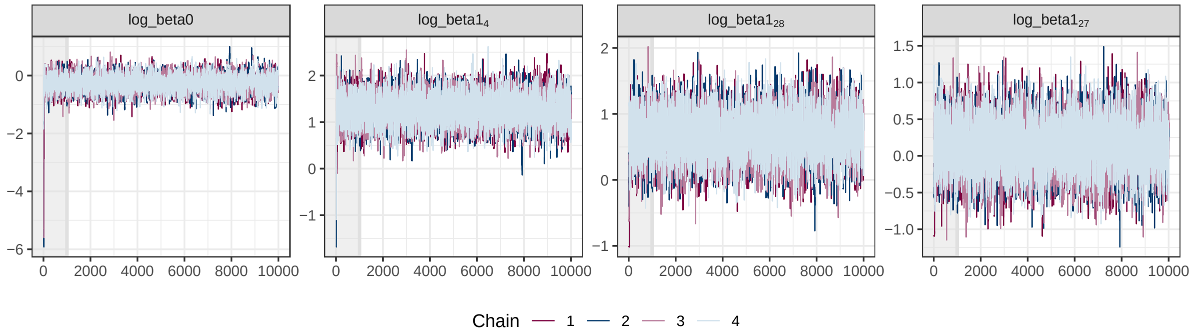

Figure 4: Trace plot of Model *ExpFt*

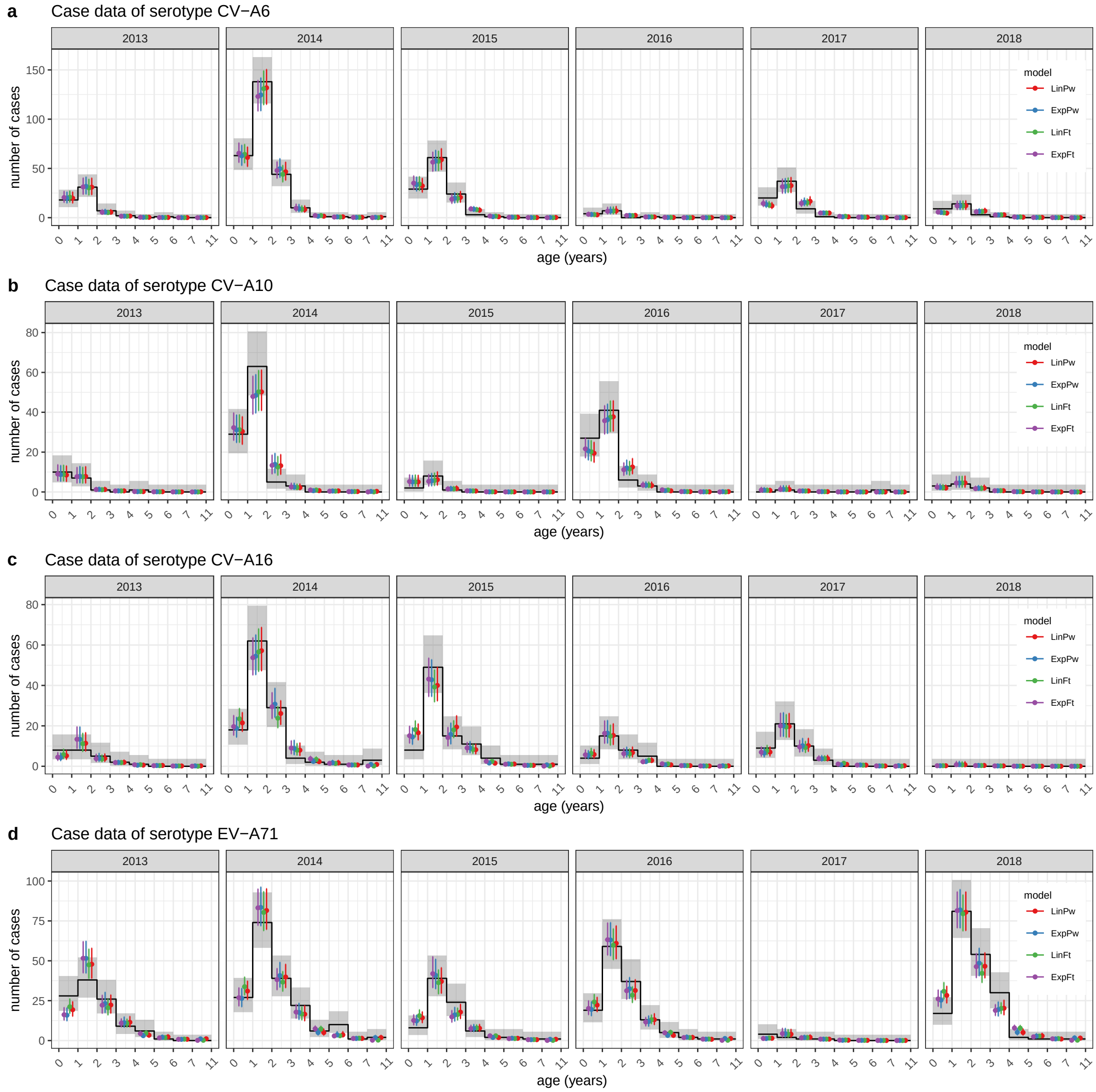

Figure 5: The reported number of cases by age and calendar year in the case data and from the estimations of 4 models. The black lines and the shaded areas are the data points in case data and the 95% confidence intervals calculated by exact method. The coloured points and the error bars are the estimations from 4 models with the 95% confidence intervals inferred from posterior distributions of the parameters. The colours correspond to 4 models. a. The estimations and data for serotype CV-A6. b. The estimations and data for serotype CV-A10. c. The estimations and data for serotype CV-A16. d. The estimations and data for serotype EV-A71.

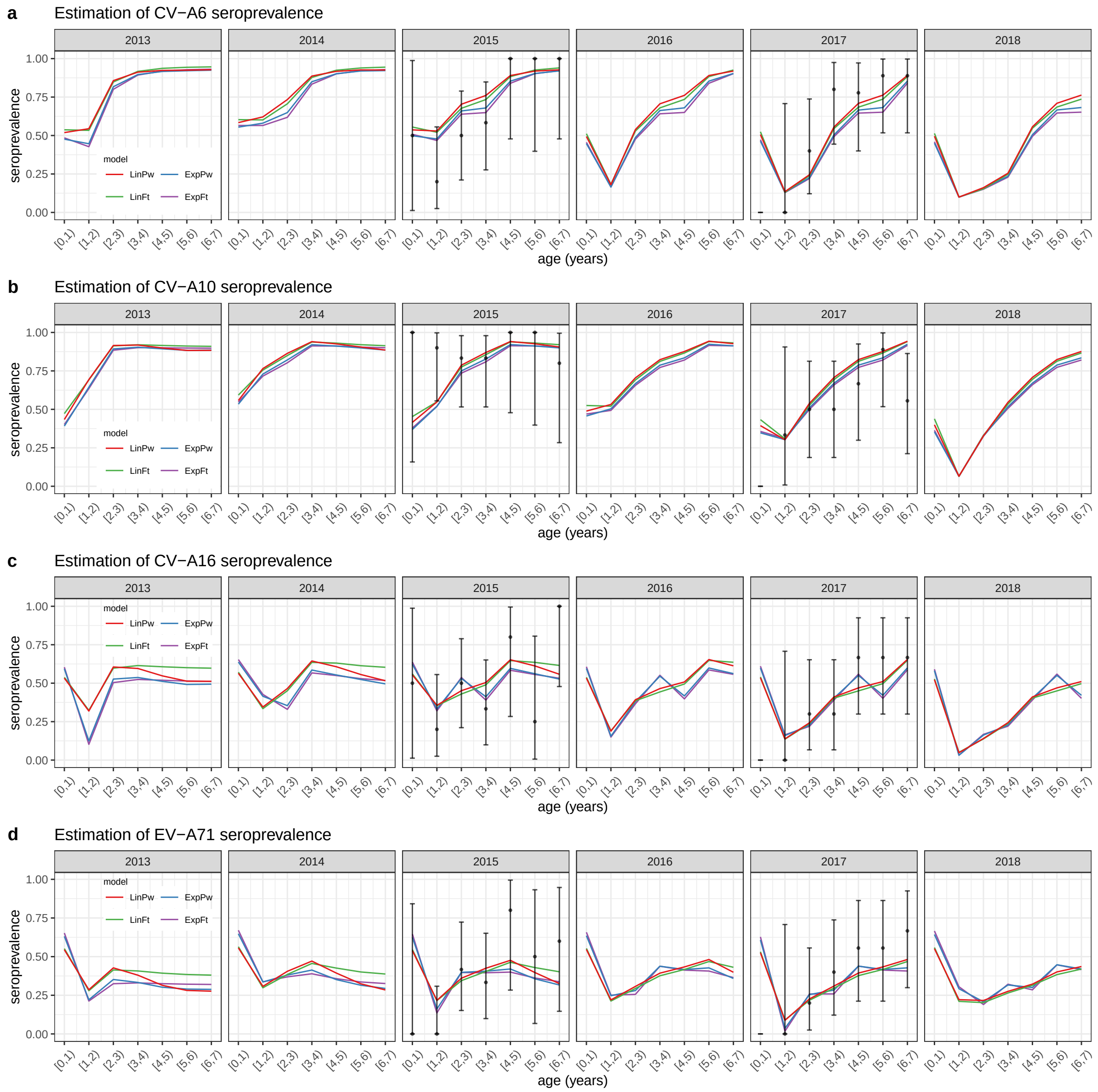

Figure 6: The seroprevalence by age, time and virus serotypes in the serological data and from the estimations of 4 models. The black points and the error bars are the data points in serological data and the 95% confidence intervals calculated by exact method. The coloured lines are the estimations from 4 models. The colours correspond to 4 models. a. The estimations and data for serotype CV-A6. b. The estimations and data for serotype CV-A10. c. The estimations and data for serotype CV-A16. d. The estimations and data for serotype EV-A71.

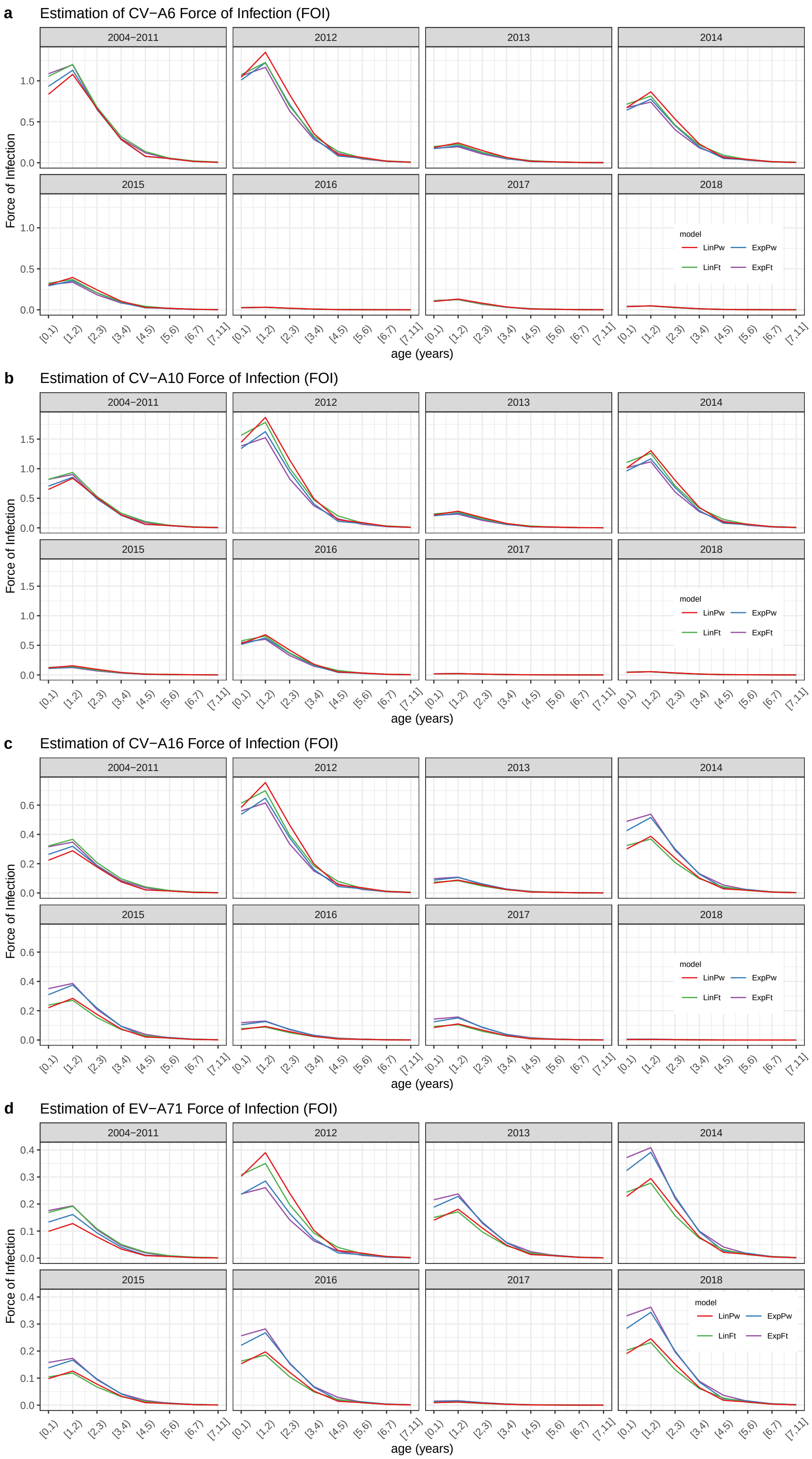

Figure 7: The estimations of age-time-specific FOI in 4 models. a. The estimations for CV-A6. b. The estimations for CV-A10. c. The estimations for CV-A16. d. The estimations for EV-A71. The colours correspond to 4 models.

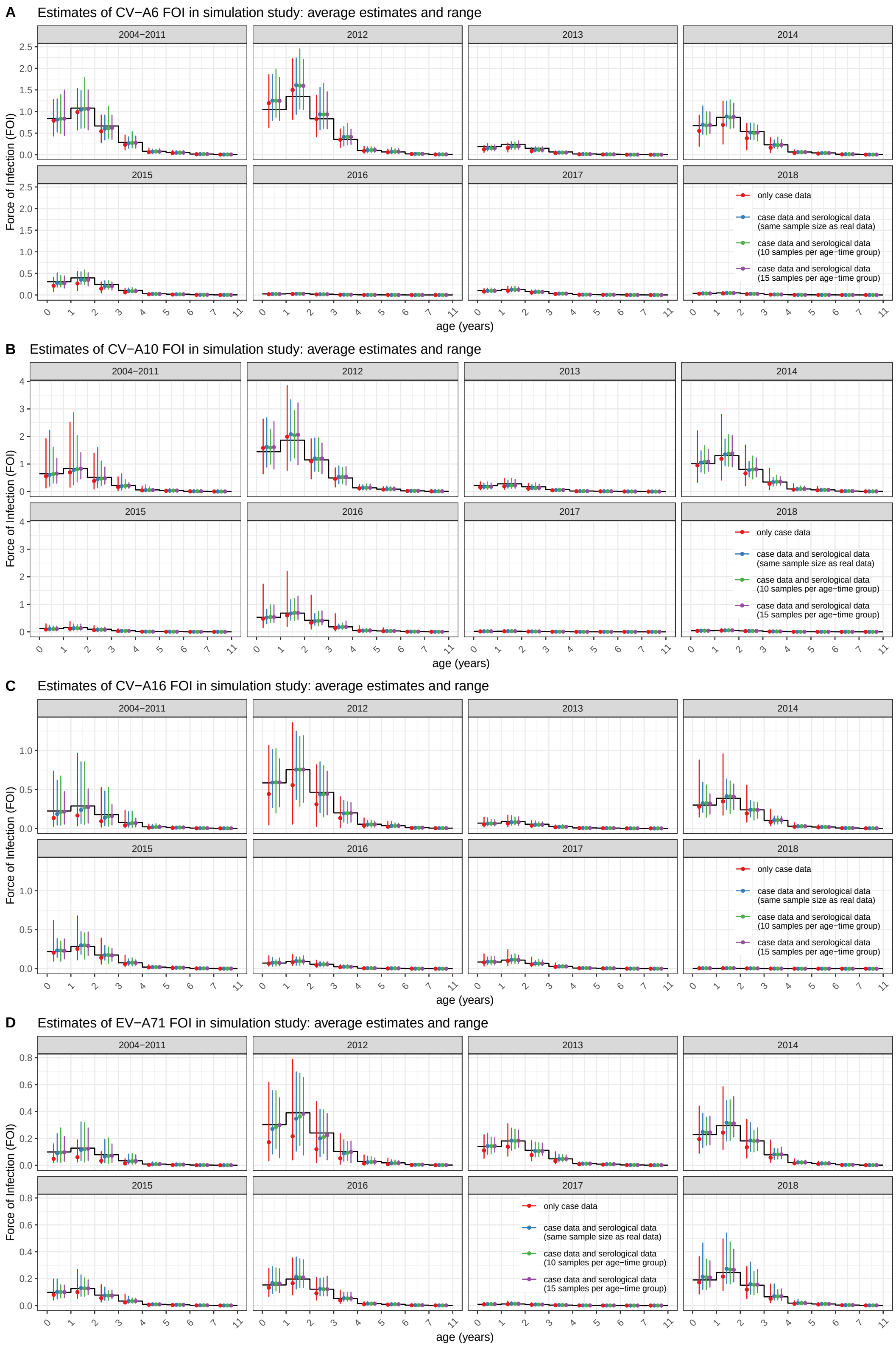

Figure 8: The average estimate and estimate range across the 100 simulated data sets for FOI parameters in simulation study. The black lines are the simulated value. The coloured points are the average estimates from models implemented on different data sets. The error bars are the corresponding estimate range. A. Estimates of FOI of serotype CV-A6. B. Estimates of FOI of serotype CV-A10. C. Estimates of FOI of serotype CV-A16. D. Estimates of FOI of serotype EV-A71.

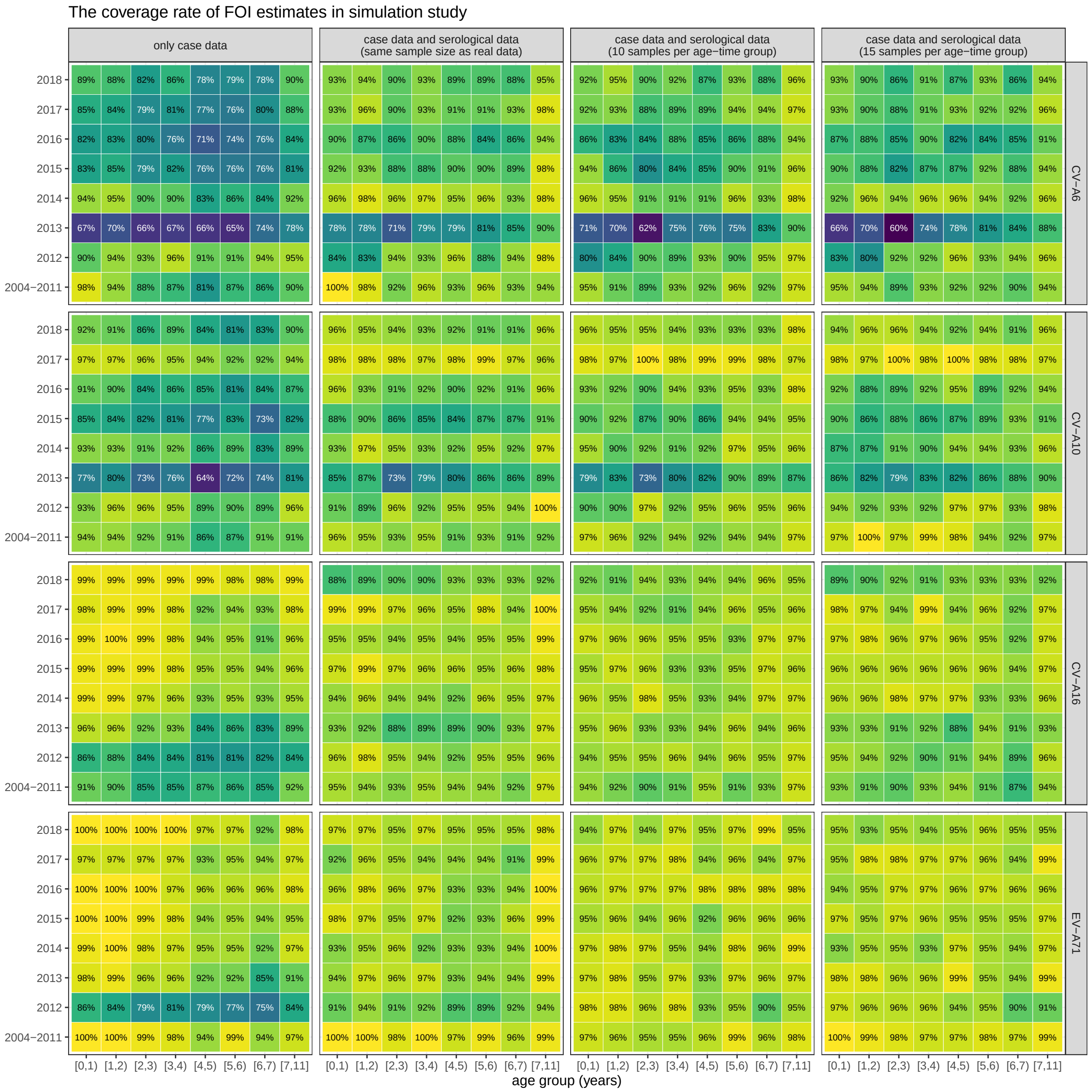

Figure 9: The coverage rate for FOI parameters in simulation study. The deeper the colour, the lower the coverage rate.

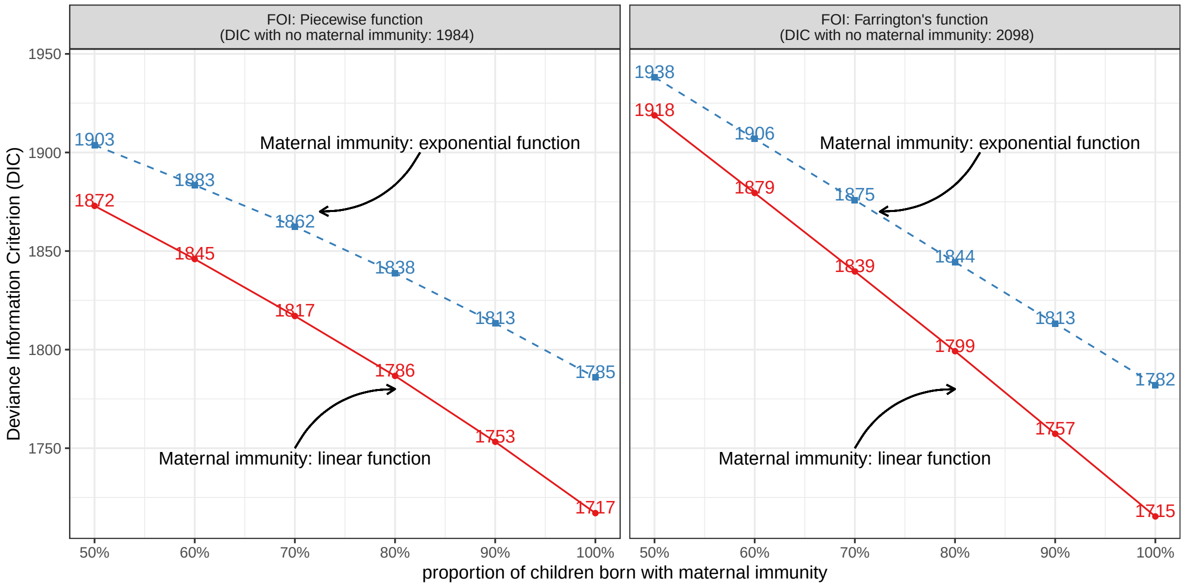

Figure 10: The value of DIC with different proportion of children born with maternal immunity for 4 model structures $LinPw$, $LinFt$, $ExpPw$, $ExpFt$, i.e., models with FOI constructed by piecewise function (Pw) or Farrington's function (Ft) and maternal immunity constructed by linear functions (Lin) or exponential functions (Exp). Smaller value of DIC indicates better model fitting effect.

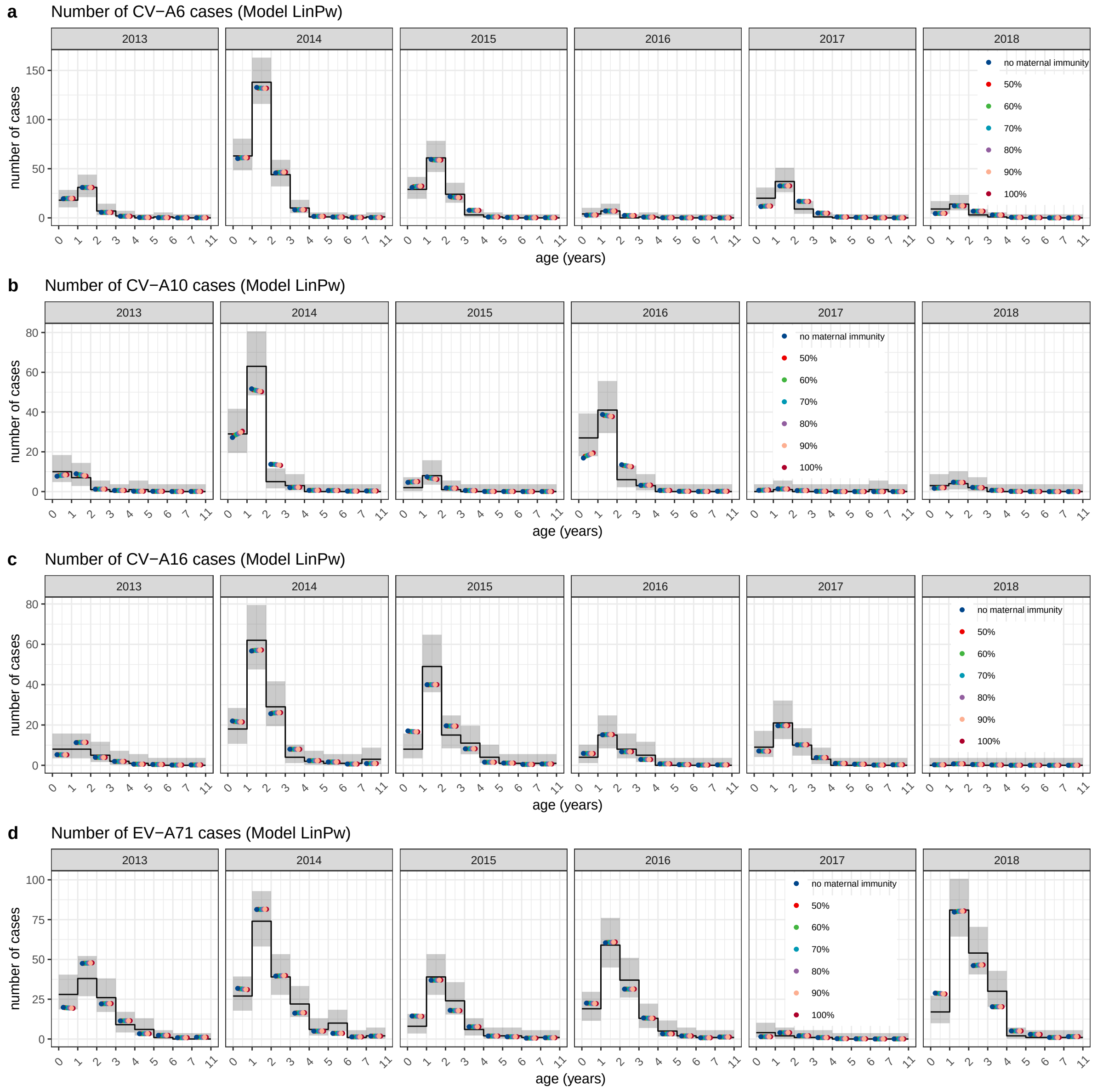

Figure 11: The estimations of case number in model *LinPw*. The coloured points are the estimations of the model. The black lines and the shaded areas are the data points and the 95% confidence intervals in the case data. a. The estimations for CV-A6. b. The estimations for CV-A10. c. The estimations for CV-A16. d. The estimations for EV-A71. The colours correspond to the proportion of children born with maternal immunity.

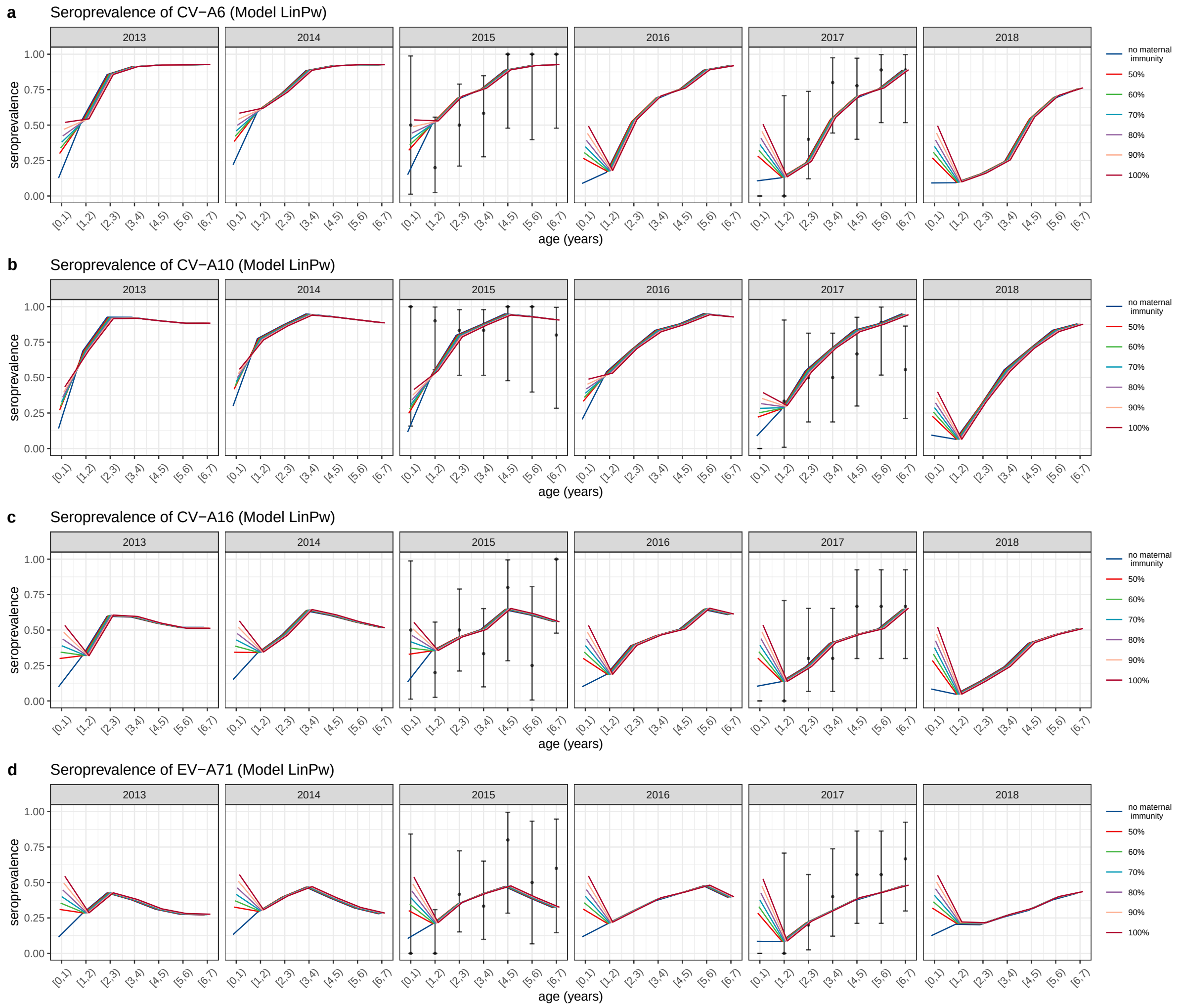

Figure 12: The estimations of seroprevalence in model $LinPw$. The coloured lines are the estimations of the model. The black points and the error bars are the data points and the 95% confidence intervals in the serological data. a. The estimations for CV-A6. b. The estimations for CV-A10. c. The estimations for CV-A16. d. The estimations for EV-A71. The colours correspond to the proportion of children born with maternal immunity.

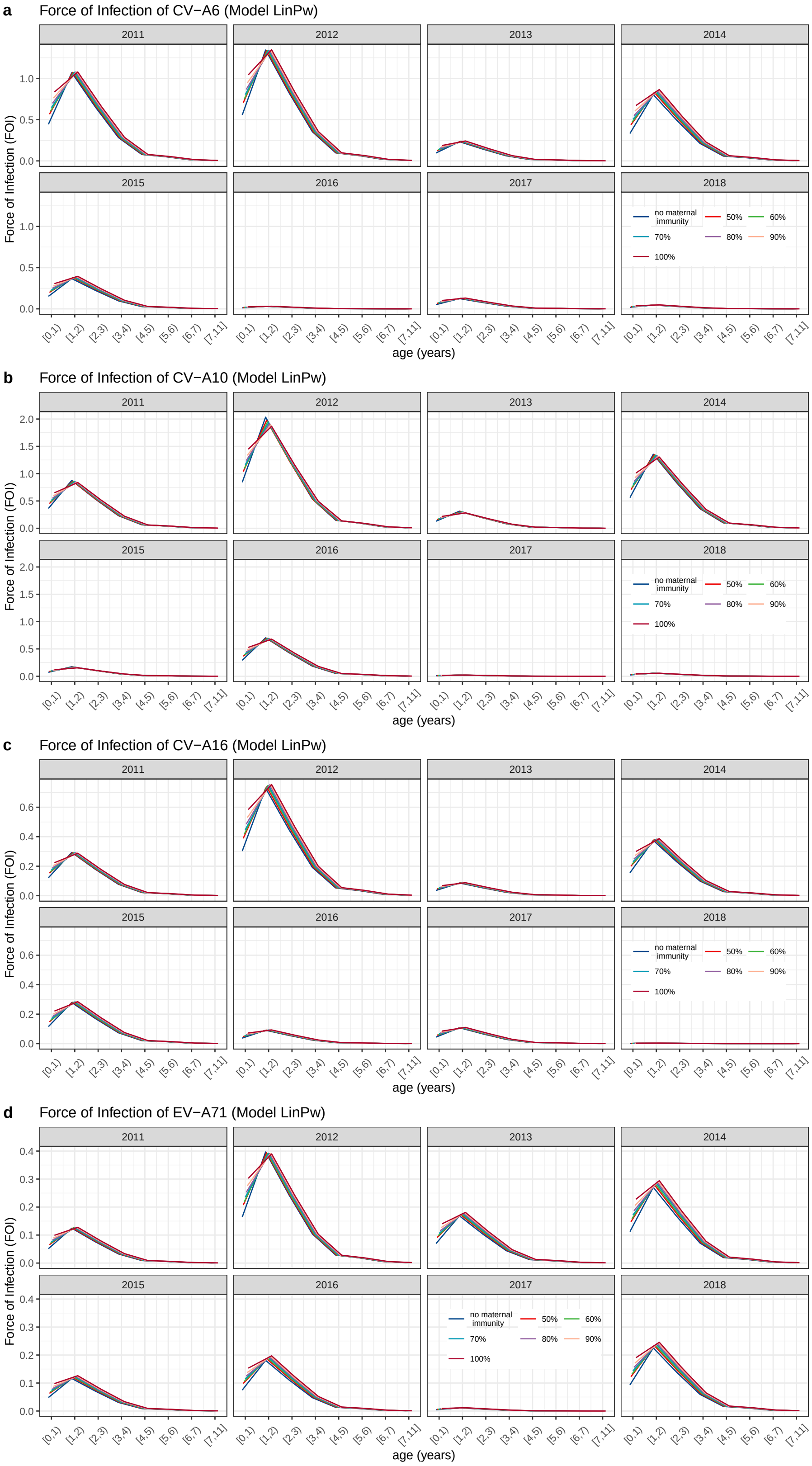

Figure 13: The estimations of age-time-specific FOI in model *LinPw*. a. The estimations for CV-A6. b. The estimations for CV-A10. c. The estimations for CV-A16. d. The estimations for EV-A71. The colours correspond to the proportion of children born with maternal immunity.

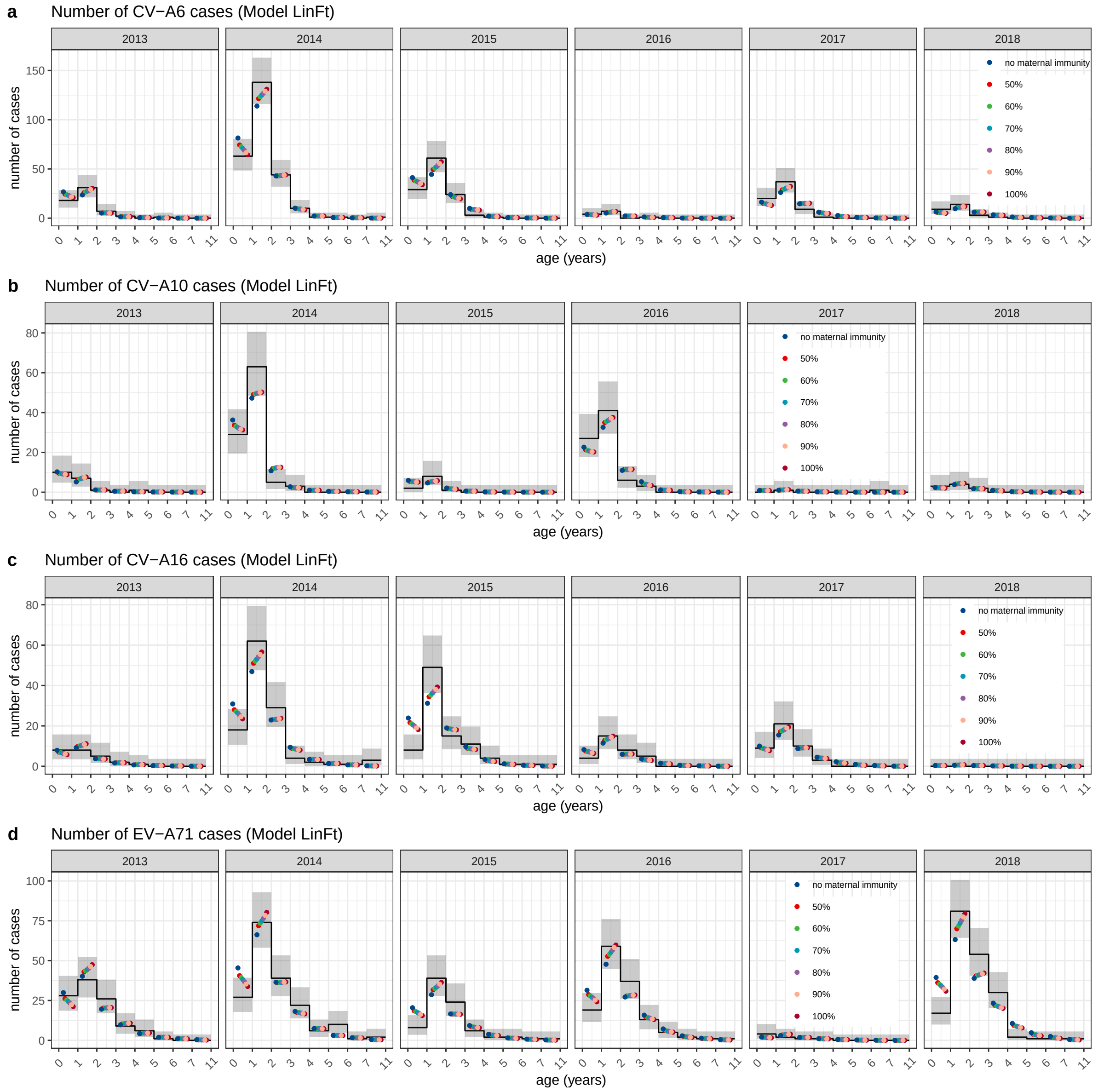

Figure 14: The estimations of case number in model *LinFt*. The coloured points are the estimations of the model. The black lines and the shaded areas are the data points and the 95% confidence intervals in the case data. a. The estimations for CV-A6. b. The estimations for CV-A10. c. The estimations for CV-A16. d. The estimations for EV-A71. The colours correspond to the proportion of children born with maternal immunity.

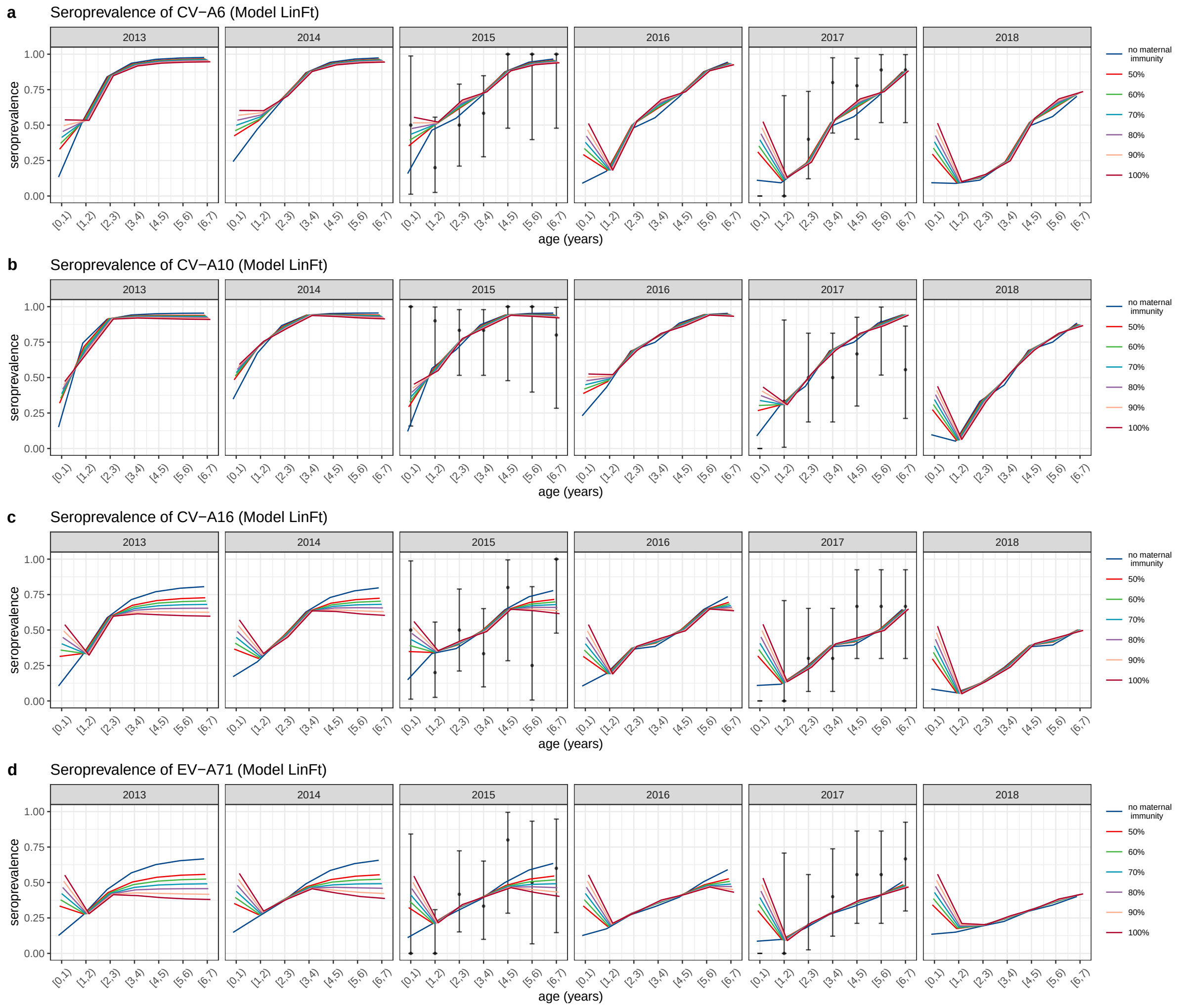

Figure 15: The estimations of seroprevalence in model *LinFt*. The coloured lines are the estimations of the model. The black points and the error bars are the data points and the 95% confidence intervals in the serological data. a. The estimations for CV-A6. b. The estimations for CV-A10. c. The estimations for CV-A16. d. The estimations for EV-A71. The colours correspond to the proportion of children born with maternal immunity.

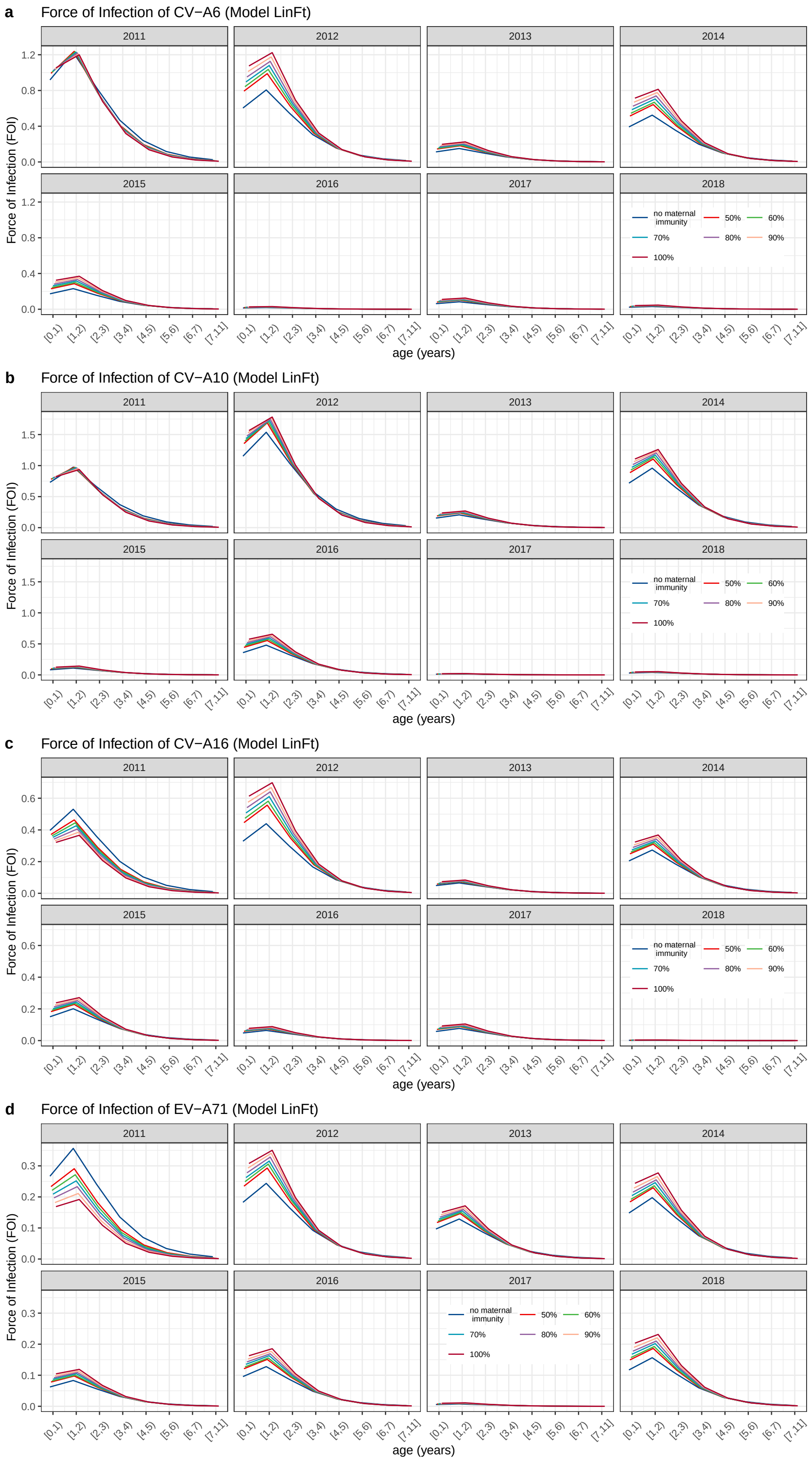

Figure 16: The estimations of age-time-specific FOI in model *LinFt*. a. The estimations for CV-A6. b. The estimations for CV-A10. c. The estimations for CV-A16. d. The estimations for EV-A71. The colours correspond to the proportion of children born with maternal immunity.

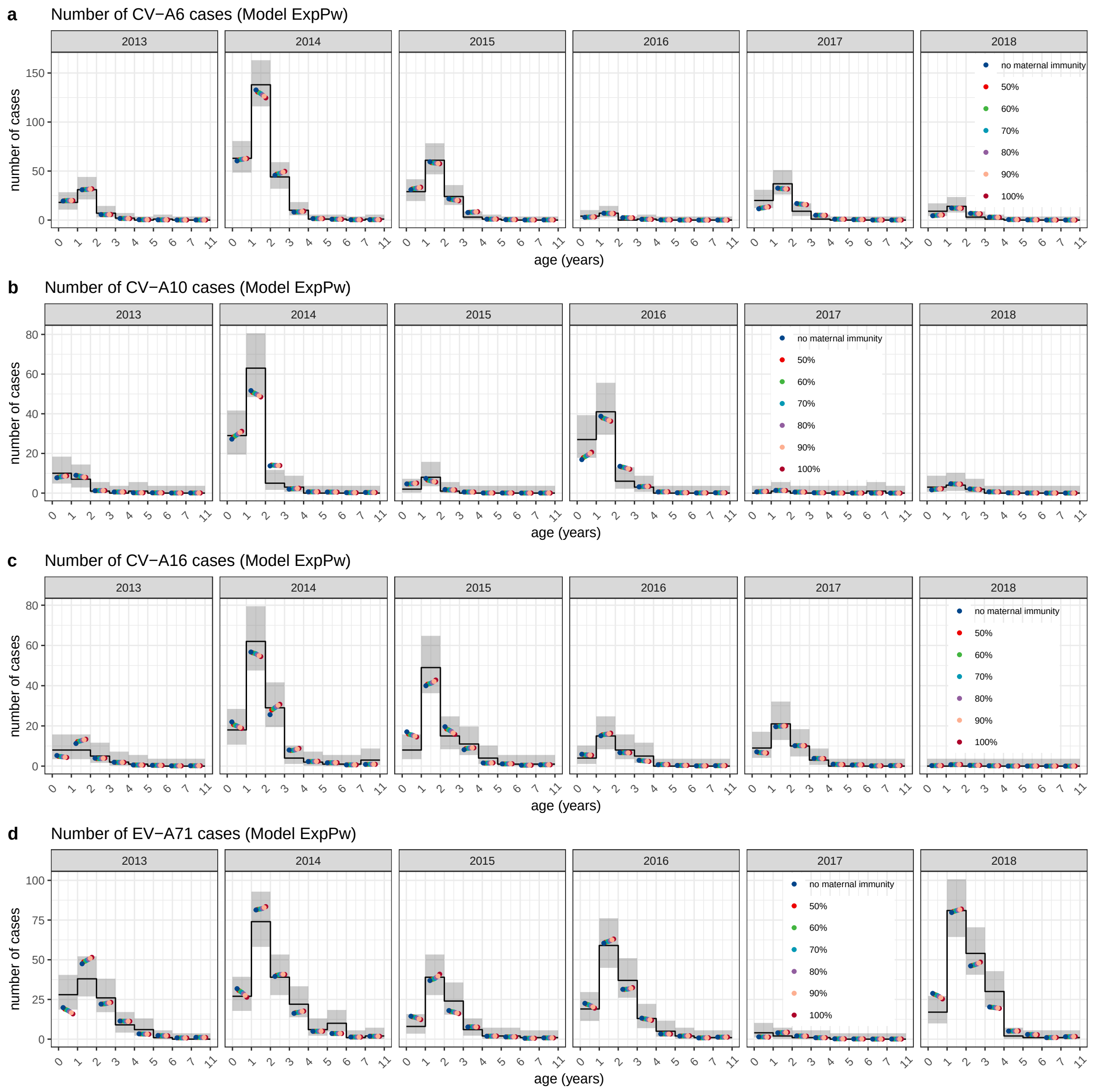

Figure 17: The estimations of case number in model *ExpPw*. The coloured points are the estimations of the model. The black lines and the shaded areas are the data points and the 95% confidence intervals in the case data. a. The estimations for CV-A6. b. The estimations for CV-A10. c. The estimations for CV-A16. d. The estimations for EV-A71. The colours correspond to the proportion of children born with maternal immunity.

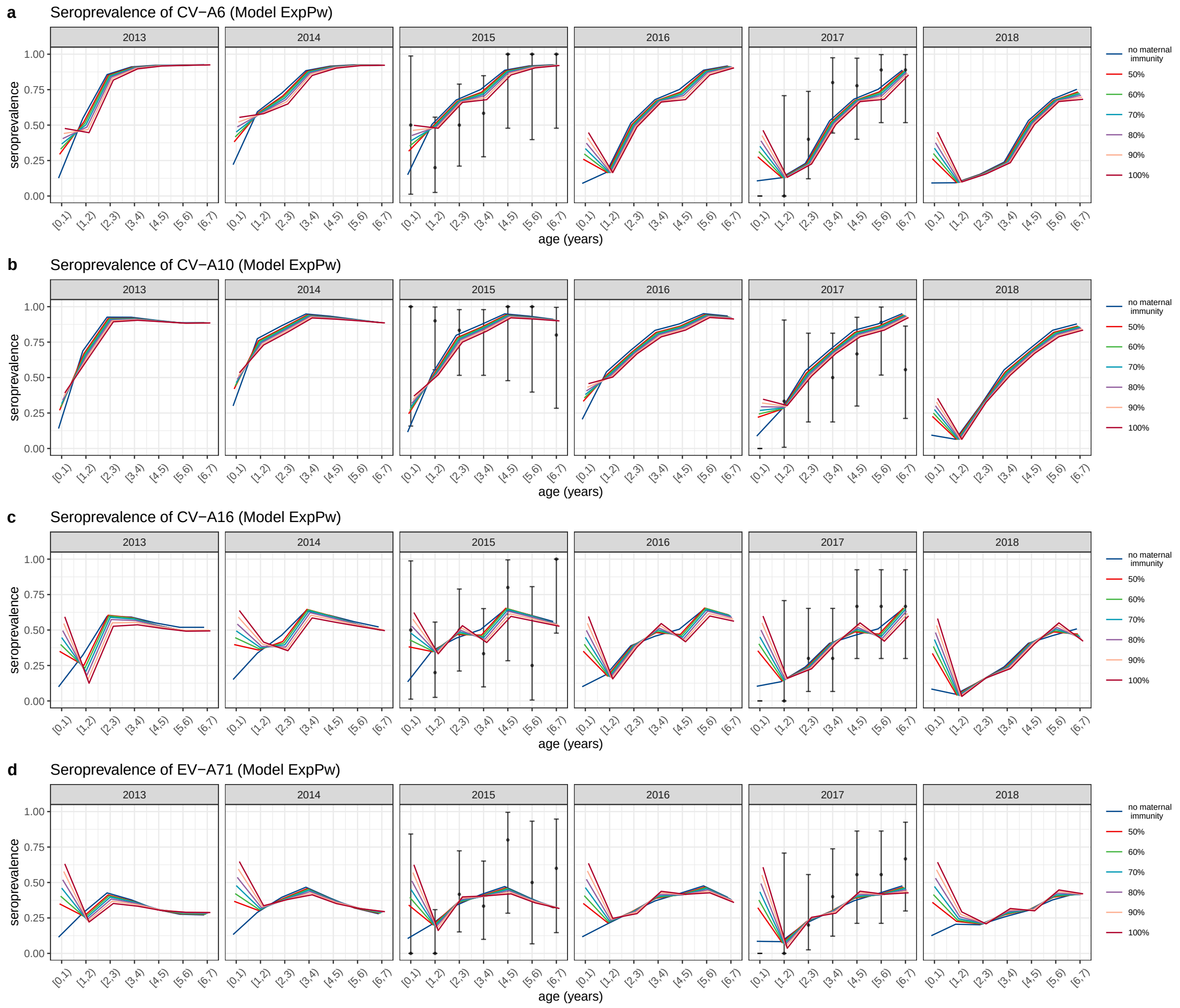

Figure 18: The estimations of seroprevalence in model *ExpPw*. The coloured lines are the estimations of the model. The black points and the error bars are the data points and the 95% confidence intervals in the serological data. a. The estimations for CV-A6. b. The estimations for CV-A10. c. The estimations for CV-A16. d. The estimations for EV-A71. The colours correspond to the proportion of children born with maternal immunity.

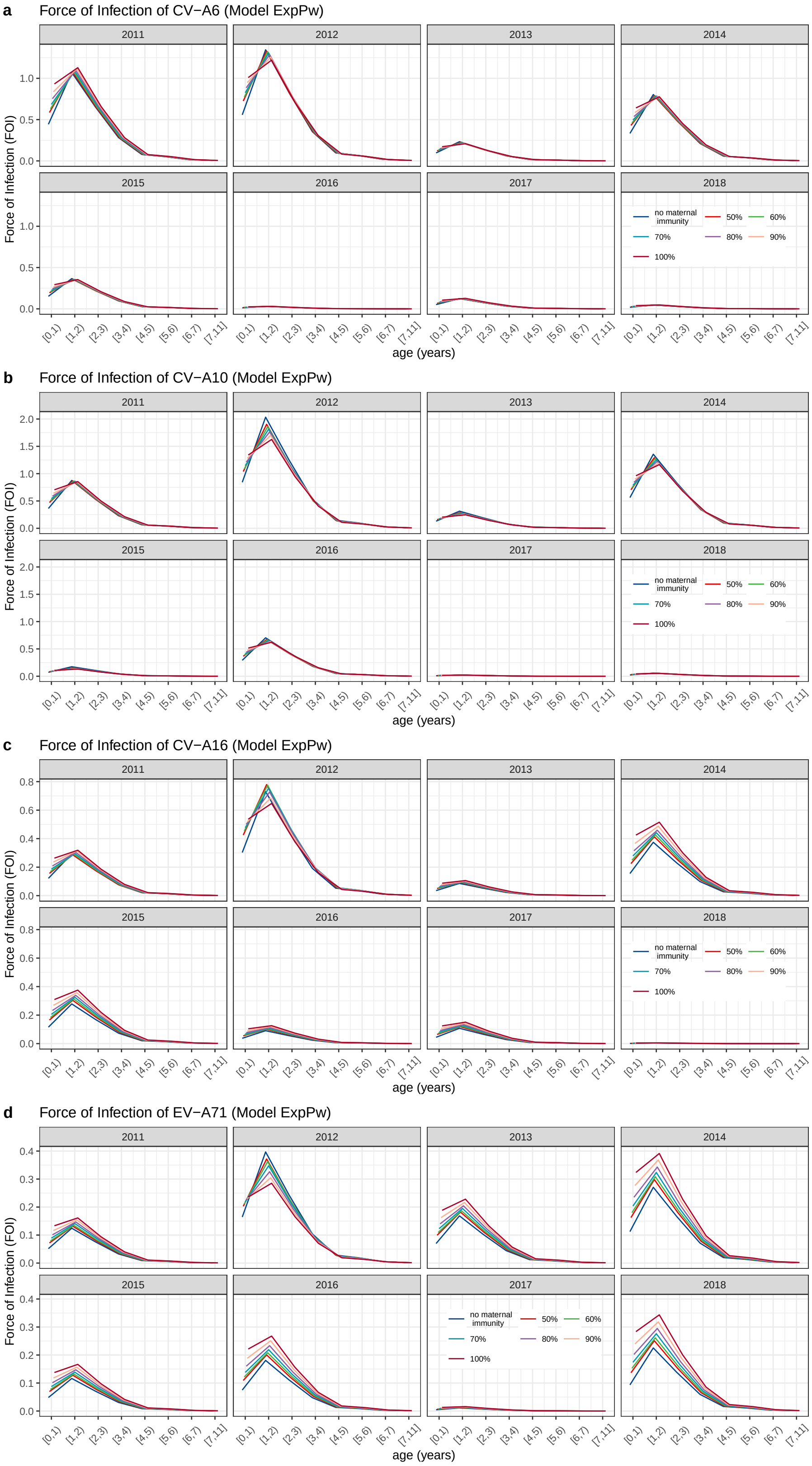

Figure 19: The estimations of age-time-specific FOI in model *ExpPw*. a. The estimations for CV-A6. b. The estimations for CV-A10. c. The estimations for CV-A16. d. The estimations for EV-A71. The colours correspond to the proportion of children born with maternal immunity.

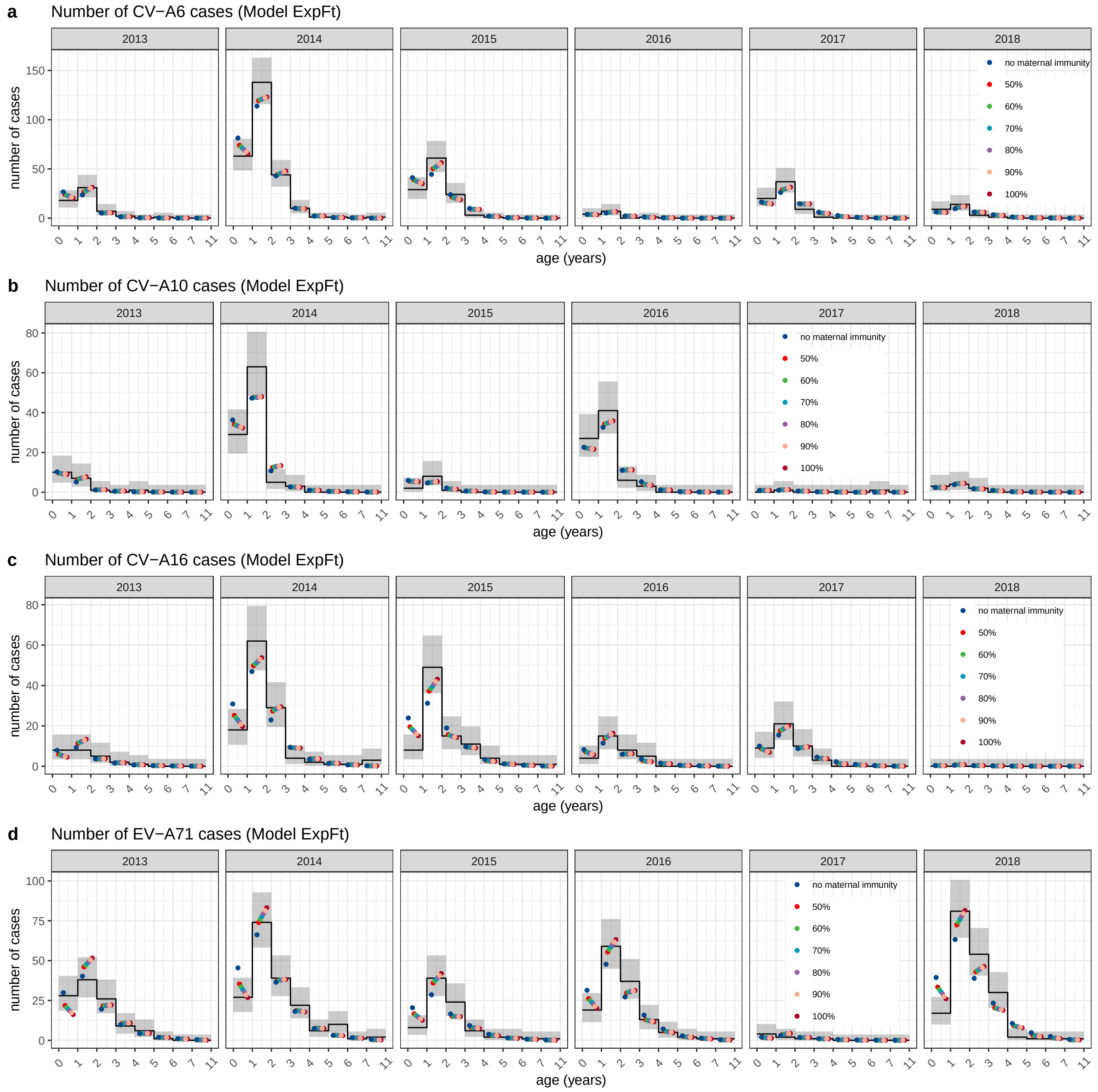

Figure 20: The estimations of case number in model *ExpFt*. The coloured points are the estimations of the model. The black lines and the shaded areas are the data points and the 95% confidence intervals in the case data. a. The estimations for CV-A6. b. The estimations for CV-A10. c. The estimations for CV-A16. d. The estimations for EV-A71. The colours correspond to the proportion of children born with maternal immunity.

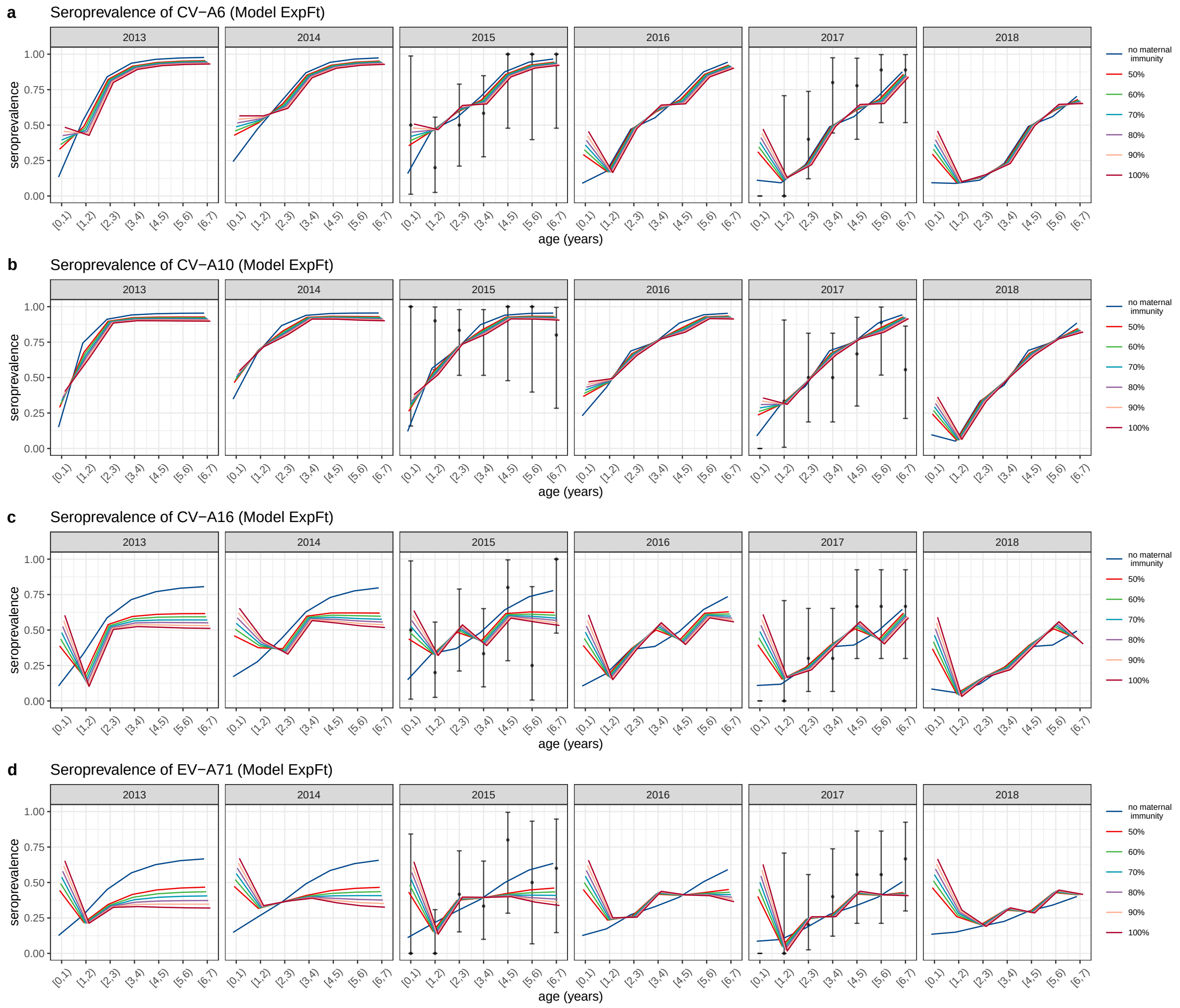

Figure 21: The estimations of seroprevalence in model *ExpFt*. The coloured lines are the estimations of the model. The black points and the error bars are the data points and the 95% confidence intervals in the serological data. a. The estimations for CV-A6. b. The estimations for CV-A10. c. The estimations for CV-A16. d. The estimations for EV-A71. The colours correspond to the proportion of children born with maternal immunity.

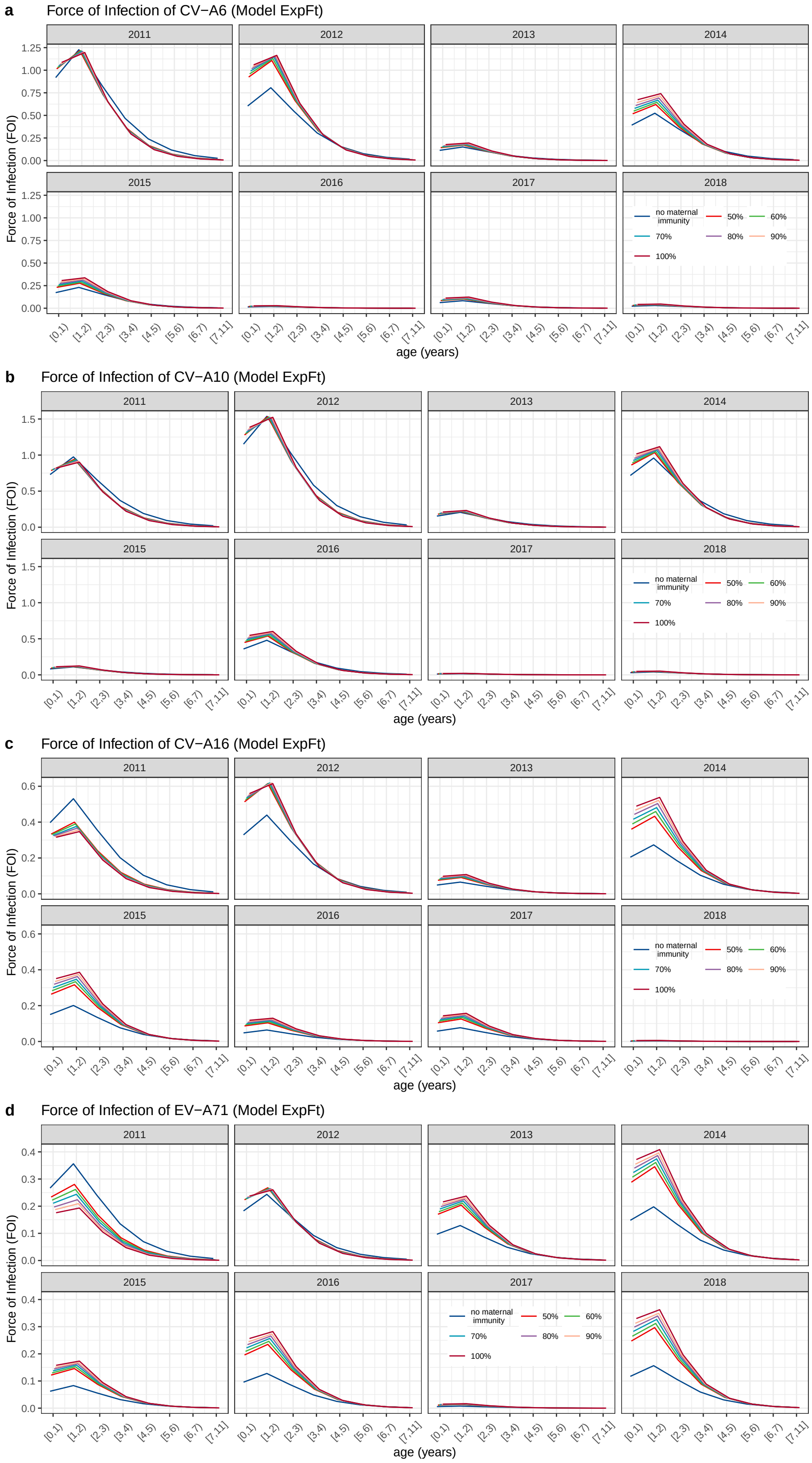

Figure 22: The estimations of age-time-specific FOI in model *ExpFt*. a. The estimations for CV-A6. b. The estimations for CV-A10. c. The estimations for CV-A16. d. The estimations for EV-A71. The colours correspond to the proportion of children born with maternal immunity.

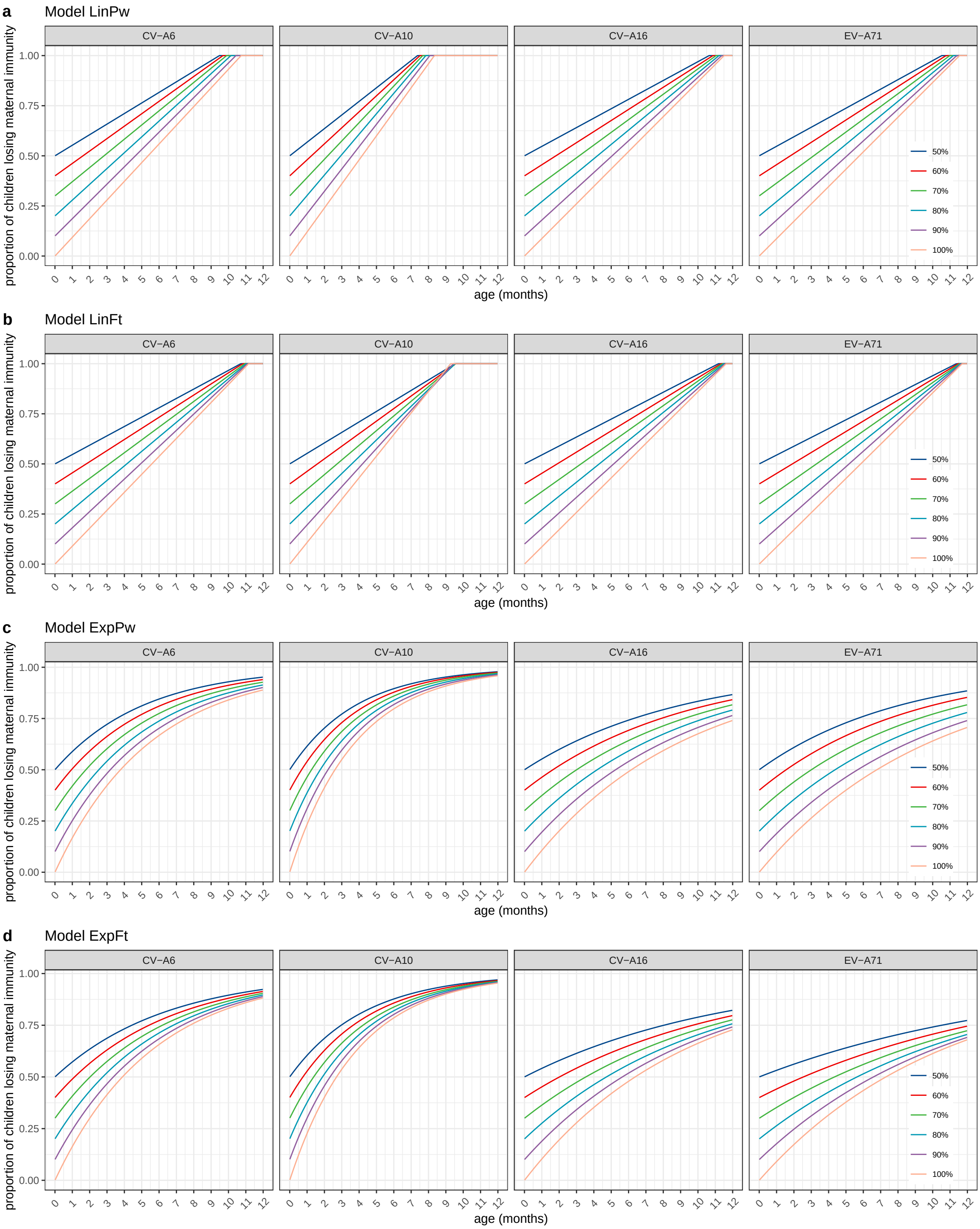

Figure 23: The proportion of children losing maternal immunity during the first year of life. a. The estimations of model *LinPw*. b. The estimations of model *LinFt*. c. The estimations of model *ExpPw*. d. The estimations of model *ExpFt*. The colours correspond to the proportion of children born with maternal immunity.

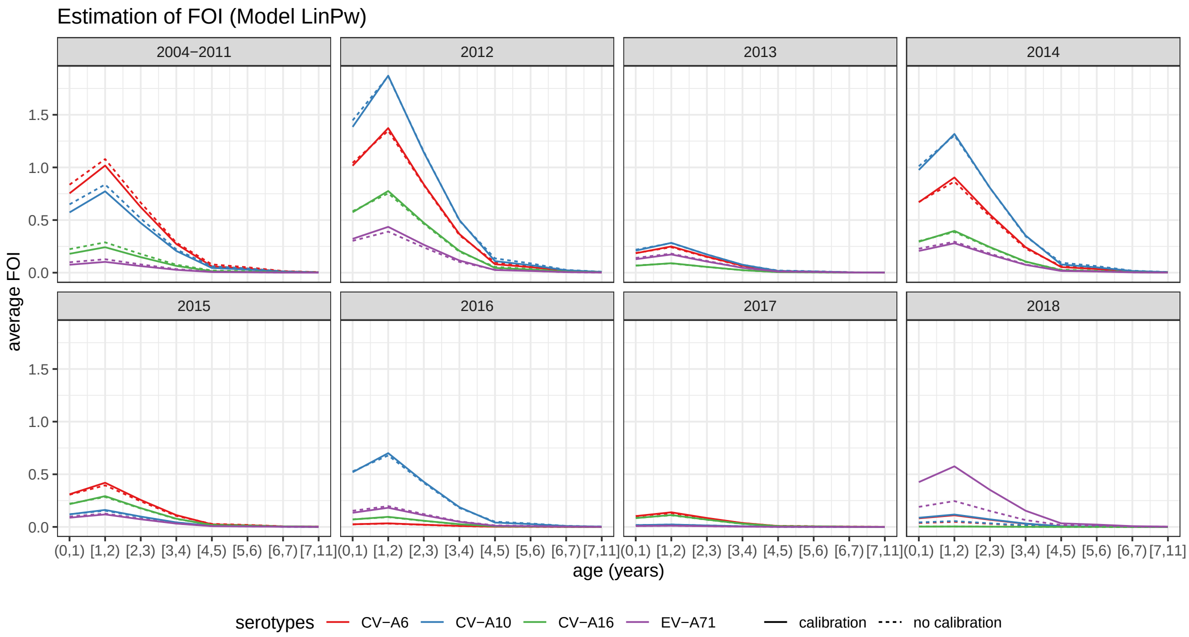

Figure 24: The estimations of age-time-specific FOI with and without calibration for case number in 2018. The colours correspond to serotypes. The solid line is the estimation based on case data with calibration. The dashed line is the estimation based on the original case data.

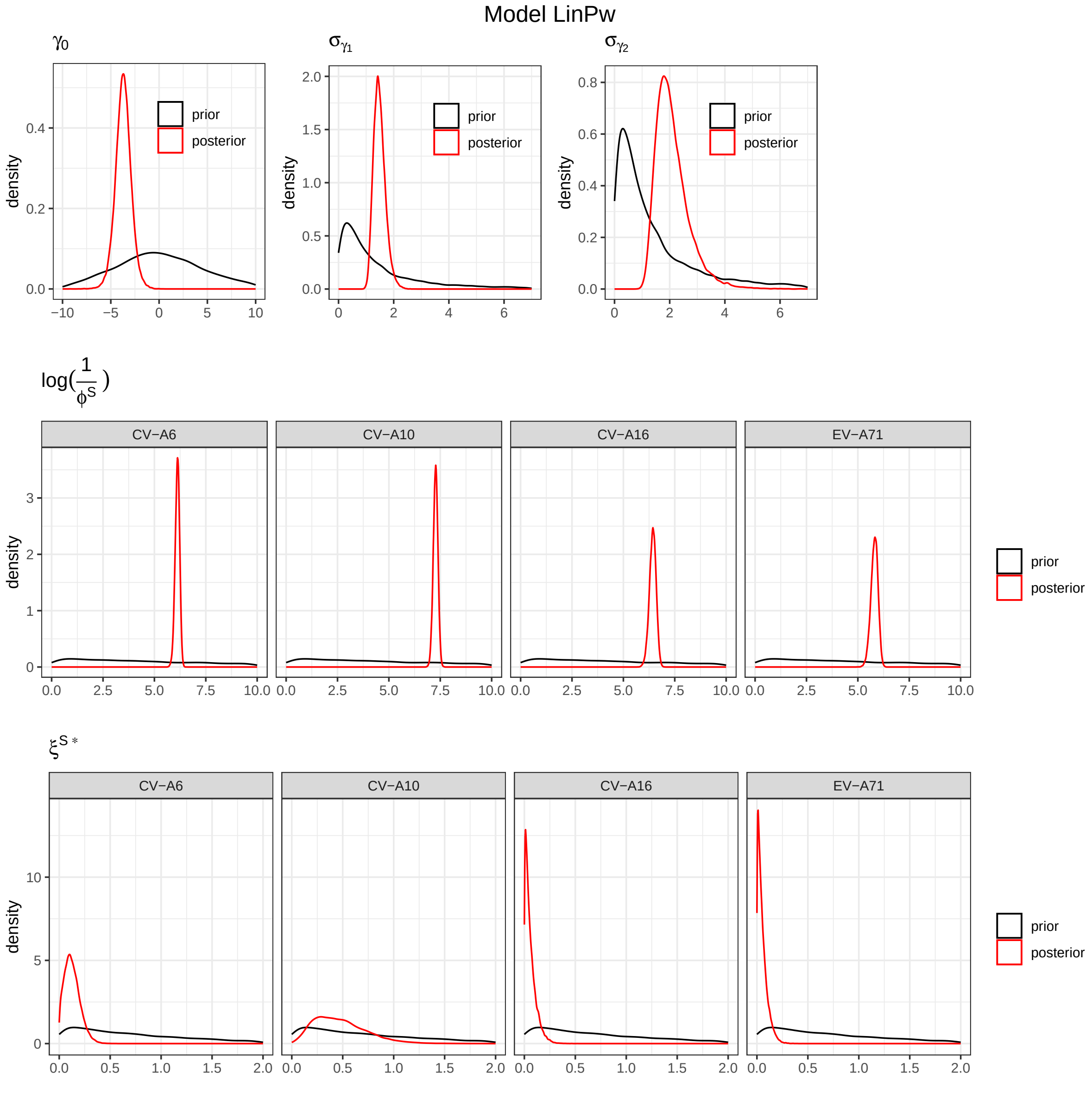

Figure 25: The density plots of prior distributions and posterior distributions in Model *LinPw*. The black lines are density of prior distributions. The red lines are density of posterior distributions.

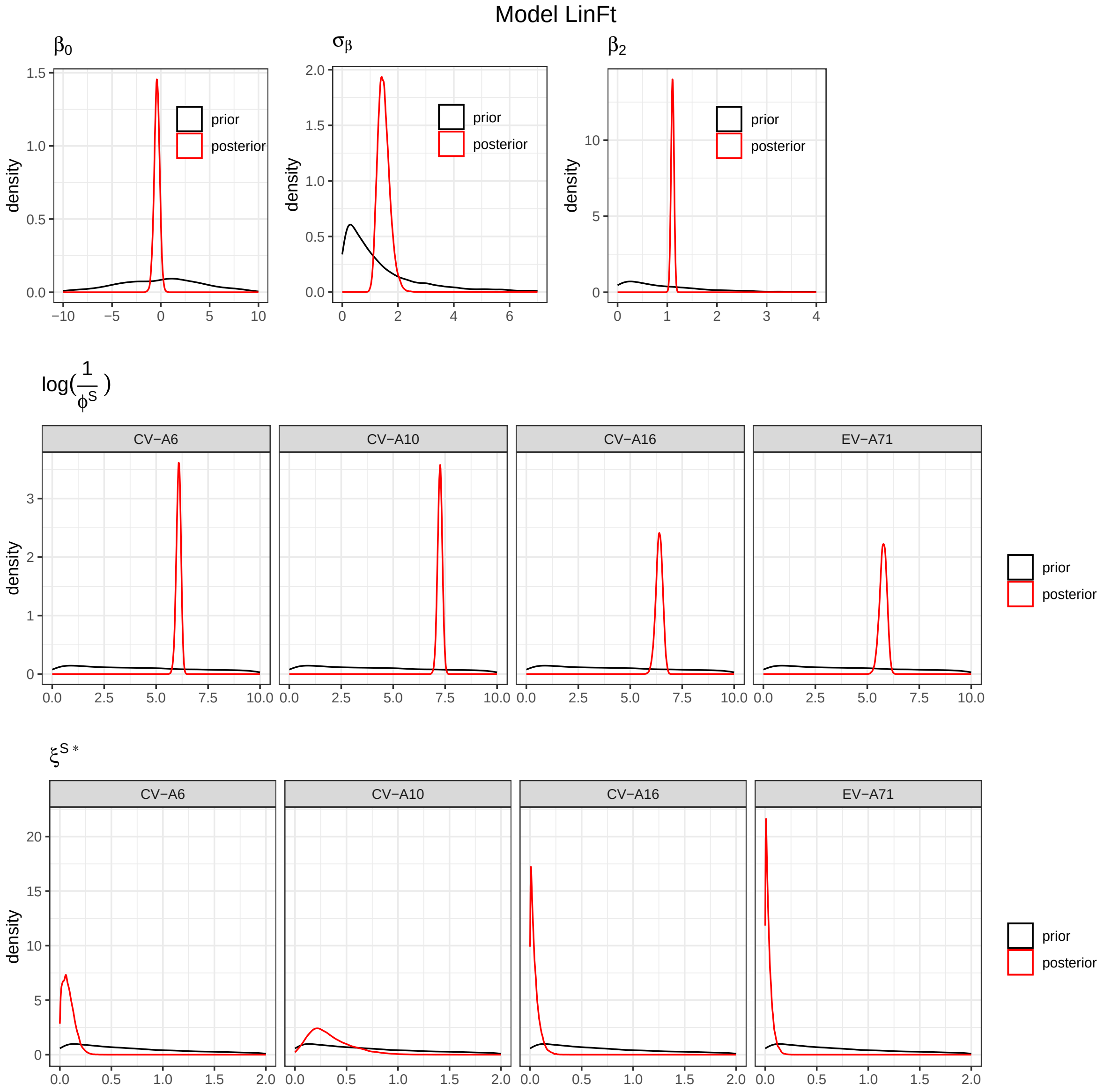

Figure 26: The density plots of prior distributions and posterior distributions in Model *LinFt*. The black lines are density of prior distributions. The red lines are density of posterior distributions.

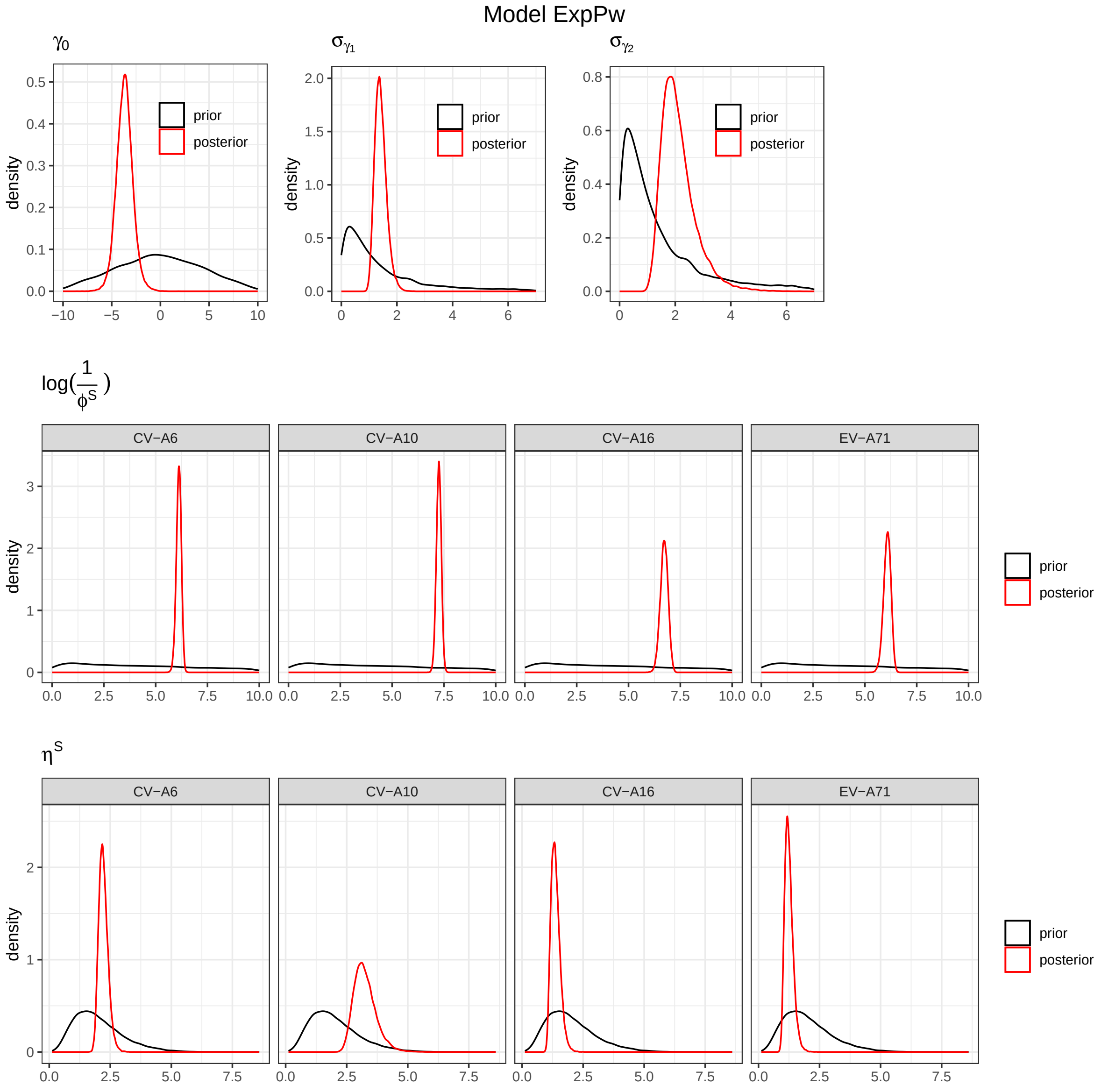

Figure 27: The density plots of prior distributions and posterior distributions in Model *ExpPw*. The black lines are density of prior distributions. The red lines are density of posterior distributions.

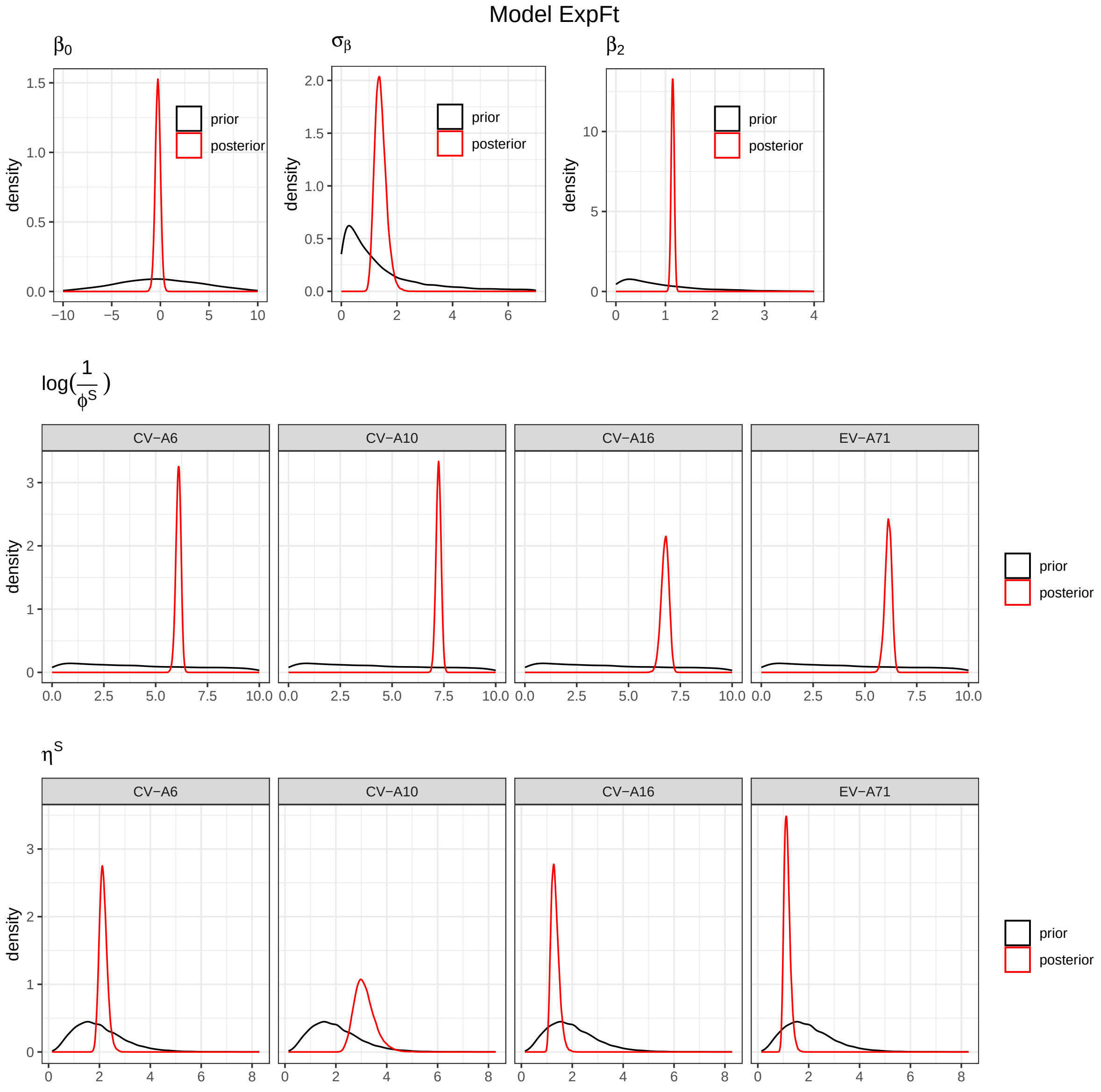

Figure 28: The density plots of prior distributions and posterior distributions in Model *ExpFt*. The black lines are density of prior distributions. The red lines are density of posterior distributions.
